# Remnants of ancestral larval eyes in an eyeless mollusk? Molecular characterization of photoreceptors in the scaphopod *Antalis entalis*

**DOI:** 10.1186/s13227-019-0140-7

**Published:** 2019-10-19

**Authors:** Tim Wollesen, Carmel McDougall, Detlev Arendt

**Affiliations:** 1 EMBL, Meyerhofstraße 1, 69117 Heidelberg, Germany; 2 Australian Rivers Institute, Griffith University, 170 Kessels Road, Nathan, QLD 4111 Australia

**Keywords:** Apical organ, Evolution and development, Gastropod, Lophotrochozoan, Mollusc, *Platynereis*, Polyplacophora, Retinal-binding domain, Spiralia, Vision

## Abstract

**Background:**

Eyes have evolved and been lost multiple times during animal evolution, however, the process of eye loss has only been reconstructed in a few cases. Mollusks exhibit eyes as varied as the octopod camera eye or the gastropod cup eye and are ideal systems for studying the evolution of eyes, photoreceptors, and opsins.

**Results:**

Here, we identify genes related to photoreceptor formation and function in an eyeless conchiferan mollusk, the scaphopod *Antalis entalis*, and investigate their spatial and temporal expression patterns during development. Our study reveals that the scaphopod early mid-stage trochophore larva has putative photoreceptors in a similar location and with a similar gene expression profile as the trochophore of polyplacophoran mollusks. The apical and post-trochal putative photoreceptors appear to co-express *go*-*opsin*, *six1*/*2*, *myoV*, and *eya*, while expression domains in the posterior foot and pavilion (posterior mantle opening) show co-expression of several other candidate genes but not *go*-*opsin*. Sequence analysis reveals that the scaphopod Go-opsin amino acid sequence lacks the functionally important lysine (K296; Schiff base) in the retinal-binding domain, but has not accumulated nonsense mutations and still exhibits the canonical G-protein activation domain.

**Conclusions:**

The scaphopod Go-opsin sequence reported here is the only known example of a bilaterian opsin that lacks lysine K296 in the retinal-binding domain. Although this may render the Go-opsin unable to detect light, the protein may still perform sensory functions. The location, innervation, development, and gene expression profiles of the scaphopod and polyplacophoran apical and post-trochal photoreceptors suggest that they are homologous, even though the scaphopod post-trochal photoreceptors have degenerated. This indicates that post-trochal eyes are not a polyplacophoran apomorphy but likely a molluscan synapomorphy lost in other mollusks. Scaphopod eye degeneration is probably a result of the transition to an infaunal life history and is reflected in the likely functional degeneration of Go-opsin, the loss of photoreceptor shielding pigments, and the scarce expression of genes involved in phototransduction and eye development. Our results emphasize the importance of studying a phylogenetically broad range of taxa to infer the mechanisms and direction of body plan evolution.

## Background

Vision is among the most important sensory modalities for bilaterian animals and it has been suggested that eyes have been independently gained and lost several times [1–4]. Simple cup-shaped eyes composed of photoreceptor cells and shading pigments probably already existed in the last common bilaterian ancestor [5]. Photoreceptor cells possess expanded surface areas to store photopigments (opsins), and while rhabdomeric photoreceptors possess microvilli on their apical surface for this purpose, ciliary photoreceptors possess surface extended cilia [5]. Similar gene expression profiles and comparisons of molecular and morphological characteristics of photoreceptors have shaped inferences of the putative ancestral organization of shared receptor cells [6, 7]. For example, recent studies suggest that the last common bilaterian ancestor possessed several opsins, including a canonical R-opsin, a non-canonical R-opsin, a C-opsin, a Go-opsin, a retinal pigment epithelium-retinal G-protein-coupled receptor/peropsin/retinochrome, and a neuropsin [8]. For the majority of bilaterians, it remains, however, unclear where these different opsins are expressed and whether given photoreceptor cells in different taxa are homologous or originated via evolutionary convergence (see [5, 7] for detailed studies).

**Fig. 1:**
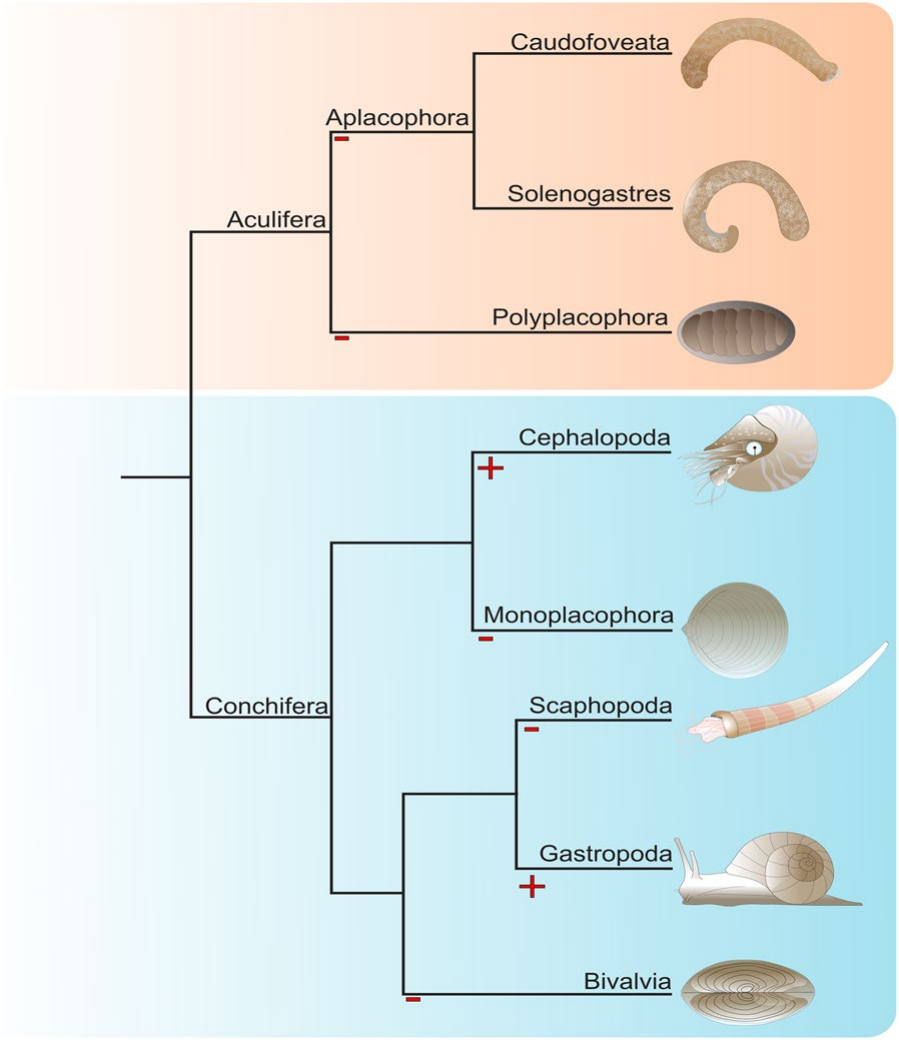
The presence and absence of cerebral eyes in adult recent mollusks. The last common cephalopod and gastropod ancestors possessed probably a pair of adult cerebral eyes (+), while the ancestors of all other molluscan clades most likely did not exhibit cerebral eyes (−). Adult polyplacophorans possess a sensory system with lenses in the tegmental layer of the shell valves, so-called esthetes. Certain bivalves evolved eyes associated with their mantle tissue. Phylogenetic analysis based on Smith et al. [28] Sketch drawing modified from Wollesen et al. [44]

Among bilaterians mollusks are textbook examples for eye evolution with designs as varied as the octopod camera eye, the nautiloid pinhole eye, the gastropod cup eye, or the camera-type eyes of strombid conchs [9]. Although most adult bivalves lack eyes, ark clams possess sophisticated compound eyes and scallops exhibit mirror-based eyes. Adult polyplacophorans lack cerebral eyes but certain species possess image-forming eyes embedded in their outermost tegmental shell valve layer (esthetes) [10]. Within earlier developmental stages, post-trochal eye spots are known from polyplacophoran trochophore larvae [10], and cerebrally innervated eyespots occur in gastropod and bivalve larvae [11]. Notably, there are also molluscan clades that neither possess eyes as adults nor during earlier developmental stages, such as the worm-shaped aplacophorans or the tusk-shelled scaphopods (Fig. 1). These enigmatic animals live an infaunal, cryptic lifestyle as adults but possess free-swimming trochophore-like larvae, in which phototactic behavior has not been reported [12–16].

Reconstructing the evolutionary history of photoreceptors is challenging within the Mollusca. A recent study showed that the polyplacophoran mollusk *Leptochiton asellus* possesses three clusters of photoreceptors located in the apical, post-trochal, and the most posterior region of its trochophore larva [17, 18]. The topography and cell lineage of the post-trochal eyes were used as arguments against their homology with cerebral eyes of other mollusks and other bilaterians [19]. Despite this, all three types of polyplacophoran photoreceptors share gene expression profiles of cerebral eyes and express photoreceptor genes such as *r*-*opsin* and *xenopsin*, as well as genes implicated in the development of cerebral eyes in other taxa [17, 18]. The latter genes include members of the *Pax*-*Six*-*Eya*-*Dach* network [*paired box protein 6* (*pax6*), *sine oculis homeobox gene 1*/*2* (*six1*/*2*), *eyes absent* (*eya*), and *dachshund* (*dach*)], transcription factors that are involved in the development of eyes, other sensory organs, and the brain [20, 21]. *Transient receptor potential cation channel* (*trpC*) is involved in phototransduction, *myosinV* (*myoV*) in intracellular r-opsin transport, while *retinitis pigmentosa GTPase regulator* (*rpgr*) is implicated in ciliary opsin targeting [22–24]. The ‘cerebral’ molecular fingerprint of polyplacophoran photoreceptors was interpreted as a heterotopic replication of the ‘cerebral eyes’ in the post-trochal region without a change in their underlying genetic circuitry [17]. According to this hypothesis, the cerebral eyes of polyplacophoran ancestors were replicated by a single saltatory event; this could be, for example, a single change in expression of a regulatory gene with concomitant changes of co-regulated downstream target genes. In the developmentally distinct post-trochal region, this would result in a pair of novel post-trochal eyes with underlying identical gene inventory to cerebral eyes. Caveats of this hypothesis are, however, that the underlying gene regulatory network is not known for polyplacophorans and that comparable data from closely related molluscan species are lacking.

In the present study, we took advantage of the case of an eyeless conchiferan mollusk, the scaphopod *Antalis entalis*, to investigate the molecular signature of eye, photoreceptor, and opsin functional degeneration. To this end, we searched for homologues of opsins and other eye or photoreceptor-related genes that have been described previously for the polyplacophoran mollusk *L. asellus* [17, 18]. Two opsin genes, *go*-*opsin* and *xenopsin*, as well as *pax6*, *six1/2*, *eya*, *dach*, *trpC*, *rpgr*, and *myosinV* have been identified to provide further insights into possible phototransduction pathways. We show that the scaphopod *A. entalis* and the polyplacophoran *L. asellus* express opsins in similar body regions and propose an evolutionary scenario of molluscan eye and photoreceptor evolution.

## Results

### Phylogenetic and sequence analysis

**Fig. 2:**
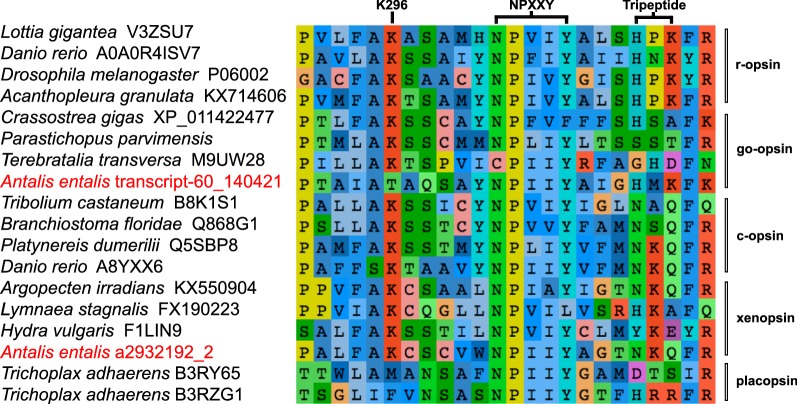
The retinal-binding domain of scaphopod Go-opsin lacks the highly conserved lysine K296. Alignment of representative sequences from Ramirez et al. [8] highlight conserved motifs as per Vöcking et al. [18]. The highly conserved lysine (K) at position 296 is absent from placozoan opsins (=‘placopsins’) and from aen-Go-opsin

**Fig. 3:**
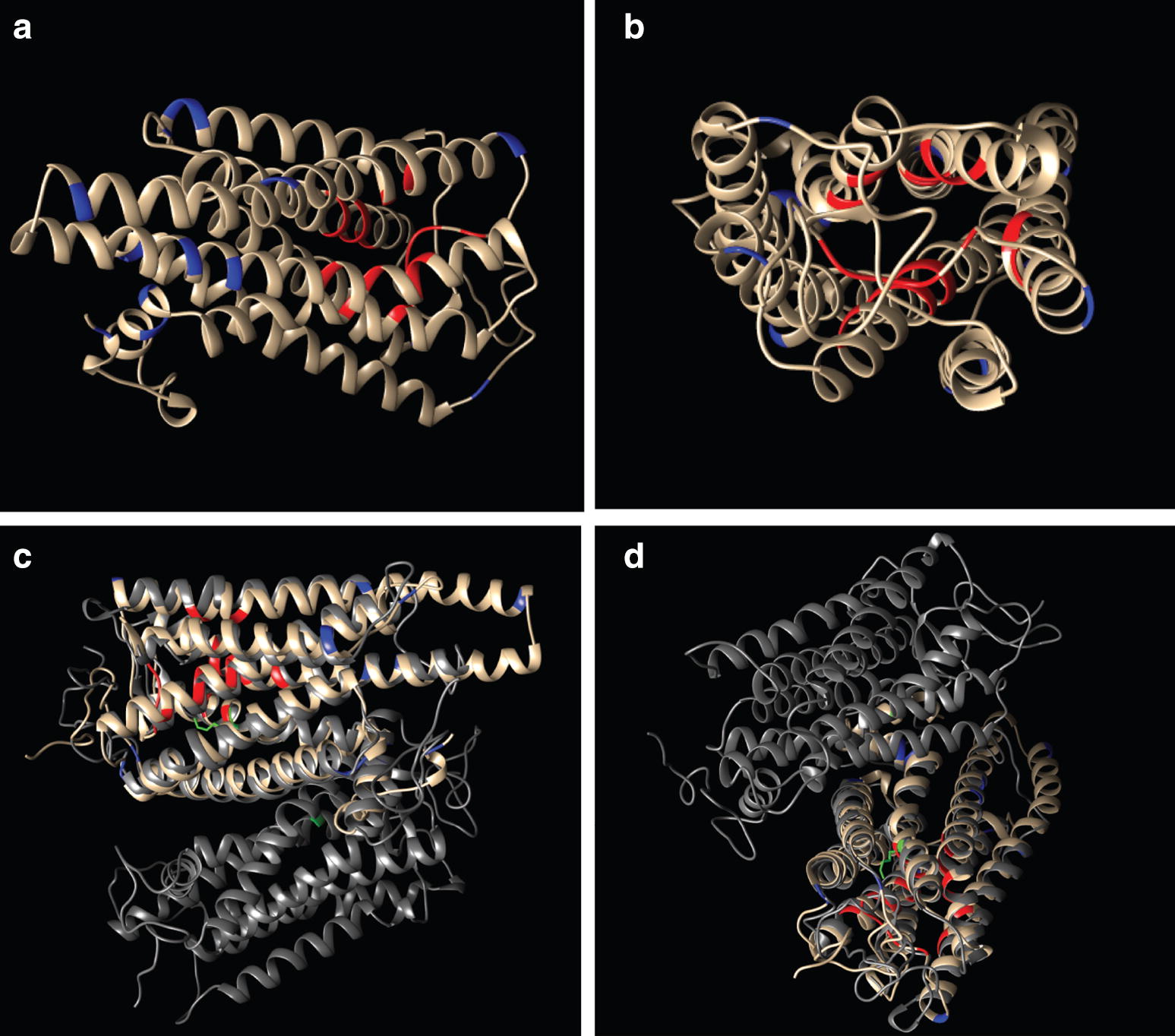
Predicted secondary structure of *Antalis entalis* Go-opsin. Lysine residues (colored blue) are not found within the retinal-binding pocket (colored red) (**a**, **b**). The predicted secondary structure of aen-Go-opsin (beige) closely aligns with that of bovine Rhodopsin (two subunits shown, gray, aen-Go-opsin is aligned with *α* subunit) (**c**, **d**). The position of K296 in the bovine subunits is indicated in green

We detected putative sequences of *go*-*opsin*, *xenopsin*, *dach*, *rpgr*, *six1*/*2*, *myosinV* (*myoV*), *pax6*, *eya*, and *trpC* within the *Antalis entalis* (*aen*) transcriptome, and the predicted protein sequences of each of these genes cluster with their bilaterian orthologs in phylogenetic analyses (Additional file 1: Figure S1). Two partial *aen* transcripts were found that encode peptides which fall within the xenopsin clade (Additional file 1: Figure S1a). The two peptide fragments do not overlap in the alignment and it is possible that they represent parts of the same gene, however, attempts to join the two fragments by PCR were unsuccessful (data not shown). We note that the Xenopsin clade is unsupported in our phylogenetic analysis, however, both partial sequences align well with other Xenopsins, and cluster with well-supported Xenopsin sequences from the more extensive analysis performed by Ramirez et al. [8]. The c-terminal Xenopsin sequence a2932192_2 was used for *aen*-*xenopsin* riboprobe synthesis for in situ hybridization. Both the c-terminal Xenopsin and the Go-opsin (*aen* transcript-60_140421) contain the characteristic ‘NPXXY’ motif and tripeptide for G-protein activation (Fig. 2; [18]). In the Xenopsin, the tripeptide is ‘NKQ’ (found in C-opsins and some other Xenopsins), while in the Go-opsin the tripeptide is ‘HMK’ (Fig. 2). The predicted amino acid sequence of *go*-*opsin* lacks the highly conserved lysine (‘K296’) in the retinal-binding domain (Fig. 2), which is completely conserved in all other opsins other than placopsins [25]. Every raw transcriptome read spanning this motif contained the same sequence, suggesting that the lack of a predicted lysine is not the result of a sequencing or assembly error (data not shown). In addition, the sequence spanning this motif has been amplified and Sanger sequenced, confirming the lack of the predicted lysine (Additional file 1). Structural prediction of *aen-*Go-opsin and comparison to bovine rhodopsin (PDB ID code 1U19) demonstrated the absence of any additional lysine residues within the retinal-binding pocket that may be able to compensate for the loss of K296 (as has been observed for Rhodopsin mutants in vitro, [26] (Fig. 3).

Clear single-copy orthologs were found for all other genes investigated, except *six1*/*2*. Three distinct *A. entalis* transcripts encoded proteins that fell within the six1/2 clade (Additional file 1: Figure S1g), suggesting that gene duplication has occurred in this lineage. The *six1*/*2* sequence most similar to the previously investigated *L. asellus six1*/*2* sequence was used for in situ hybridization.

No orthologs of R-opsin and C-opsin were identified in transcriptomes of developmental stages and adults of *A. entalis* ([27]; NCBI bioproject PRJNA357466; assembly available at https://zoology.univie.ac.at/open-data; [28], NCBI BioProject PRJNA72139).

### Gene expression analyses

In situ hybridization experiments with riboprobes against *trpC* and *xenopsin* did not yield labeling of transcripts in any of the developmental stages examined (data not shown). PCR products were amplified from pooled larval cDNA, confirming that the genes are expressed during development, but perhaps at a concentration too low to be detected by hybridization techniques.

**Fig. 4:**
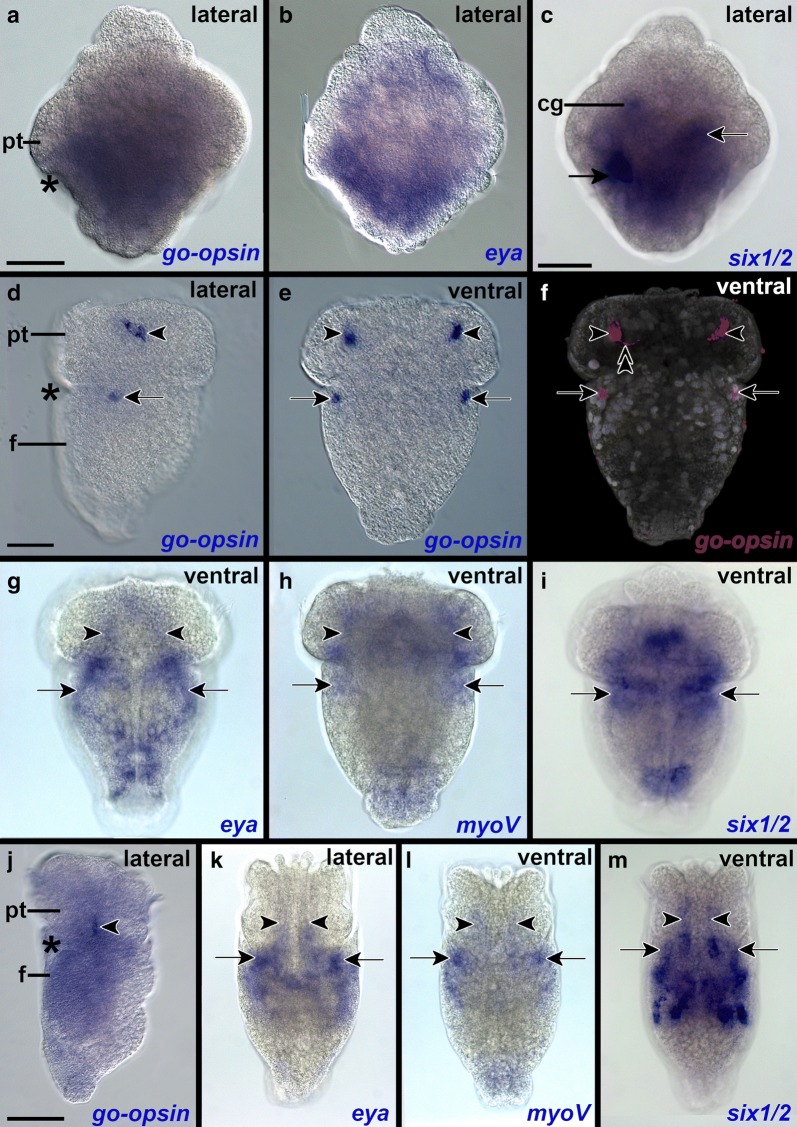
*Go*-*opsin*+ cells and potentially co-expressed genes in developmental stages of the scaphopod *Antalis entalis.* Anterior faces up in all aspects. Early trochophores (**a**–**c**) express *go*-*opsin* in the region around the mouth (asterisk), while *eya*+ cells are distributed throughout the interior of the larva. *Six1*/*2*+ cells are located in the region of the foot (black arrow), in the prospective cerebral ganglia (cg), and in two other cells (white-lined arrow). In early mid-stage trochophores (**d**–**i**) *go*-*opsin*+ cells are located in the inner anterolateral mantle margin (arrows). Two other *go*-*opsin*+ cells are located in the lateral episphere adjacent to the trochoblasts (arrowheads). **f** Axons of the apically located *go*-*opsin*+ cells run to the neuropil of the apical organ (double-arrowhead) and processes of these cells penetrate the epidermis and are in contact with the environment (not shown here, only visible in the confocal stack). Each arrowhead labels a *go*-*opsin*+ cell. **g** Putative co-expression of *eya* with both pairs of *go*-*opsin *+ cells. **h** Putative co-expression of *myoV* with both pairs of *go*-*opsin *+ cells. **i** Putative co-expression of *six1*/*2* with the posterior pair of *go*-*opsin*+ cells. In mid-stage trochophores (**j**–**m**), *go*-*opsin*, *eya*, *myoV* and *six1/2* are probably co-expressed in both apical cells that migrated in posterior direction (arrowheads) and in both post-trochal cells that are located in the anterolateral inner mantle margin (white-lined arrows). Both apical *six1*/*2*+ cells (arrowheads) are probably different cells than the *go*-*opsin*+, *eya*+, *myoV*+ cells since they derive from a different location at earlier stage (c.f. Additional file 1: Figure S8f). See Additional file 1: Figures S2, S4, S5 and S8 for a detailed description of the expression domains. *f* foot *pt* prototroch. Scale bars: 50 µm for image of each developmental stage

The earliest expression patterns detected in developmental stages of the scaphopod *Antalis entalis* are found in early trochophore larvae (Fig. 4a–c; Additional file 1: Figures S2–S8a–c). In the episphere, *pax6*+ cells are located below the cerebral pits, i.e., the invaginations of the cerebral ganglia placodes (Additional file 1: Figure S6a–c; [29–31]). Close to the *pax6*+ cells, *six1*/*2*+ cells are situated that may also be associated with the nervous system (Fig. 4c; Additional file 1: Figure S8a–c). More posteriorly *dach* is expressed in two cells close to the foregut (Additional file 1: Figure S3a–c), while two *myoV*+ cells are located at the level of the prototroch (Additional file 1: Figure S5a–c). *Go*-*opsin* is co-expressed with *eya*, *six1*/*2*, and *rpgr* posterior to the prototroch (Fig. 4a–c; Additional file 1: Figures S2a–c, S4a–c, S7a, S8a). Posterior to the mouth *dach* is expressed in two bilateral cells embedded in the epidermis (Additional file 1: Figure S3a–c). A pair of *pax6*+ cells is located in the prospective foot, a region in which *eya*+ cells and *dach*+ cells are also present (Fig. 4b; Additional file 1: Figures S3a–c, S4a–c, S6a–c).

**Fig. 5:**
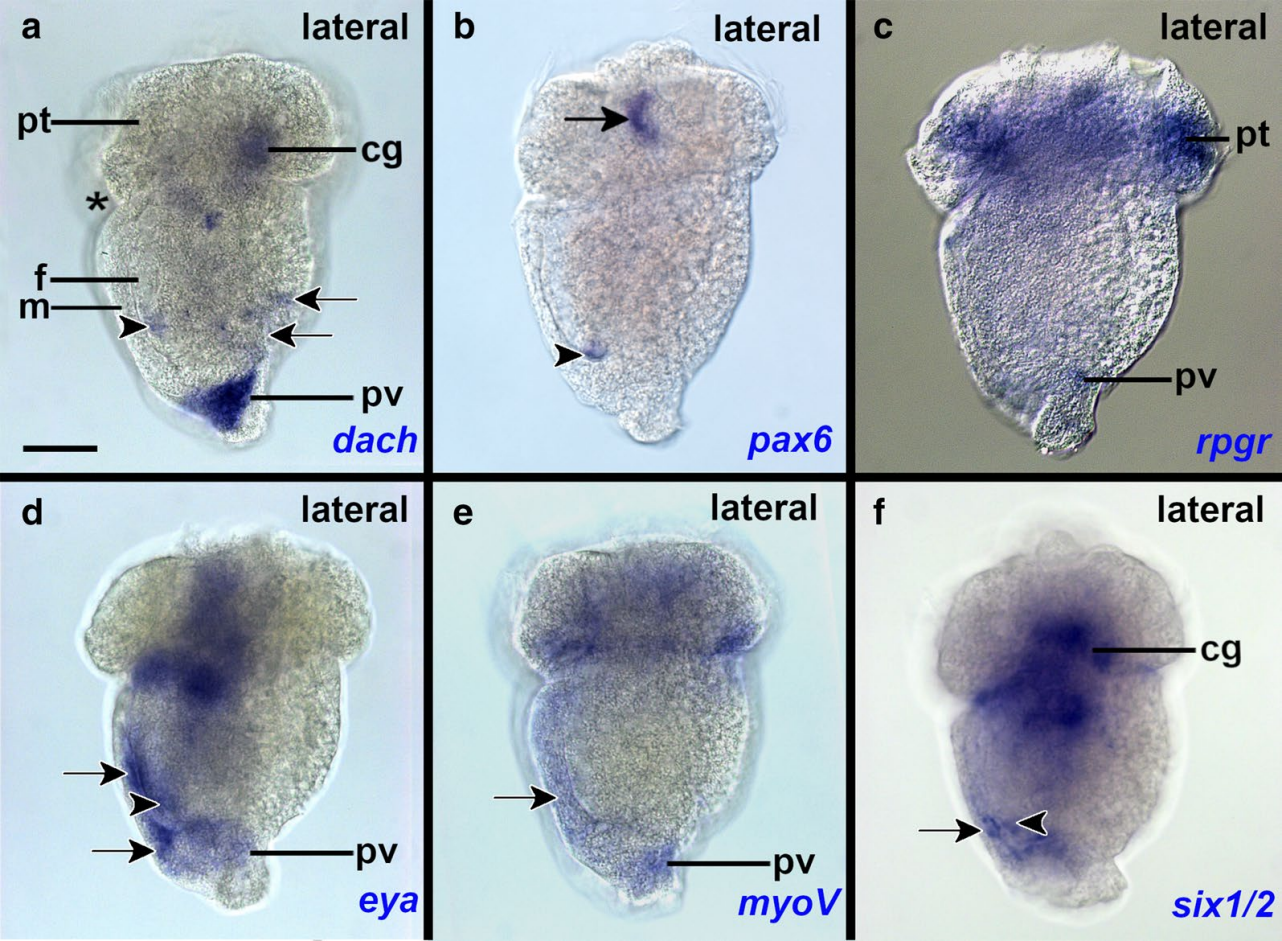
Putative sensory epithelia in the hyposphere of the scaphopod trochophore. Anterior faces up and ventral to the left in all aspects. See Fig. 4 for description of other expression domains of respective genes. The asterisk labels the mouth. **a** *Dach* is expressed in the region of the cerebral ganglia (cg), the pavilion (pv), the anterolateral and posterior foot (arrowhead), the posterior dorsal mantle region (arrows), and the lateral foot. **b** Two *pax6*+ flask-shaped cells are part of the lateral apical organ (black arrow) and two *pax6*+ cells are located in the posterior foot (white-lined arrowhead). **c** *Rpgr* is expressed in the trochoblasts of the prototroch and cells of the pavilion. **d** *Eya* is expressed in cells of the pavilion, the posterior ventral mantle (white-lined arrows), and the posterior foot (arrowhead). **e** *MyoV*+ cells are located in the ventral mantle (arrow) and the pavilion. **f** *Six1*/*2*+ cells are located in the posterior ventral mantle (arrow) and the posterior foot (arrowhead) as well as the inner pavilion. *pt* prototroch. Scale bars: 50 µm

**Fig. 6:**
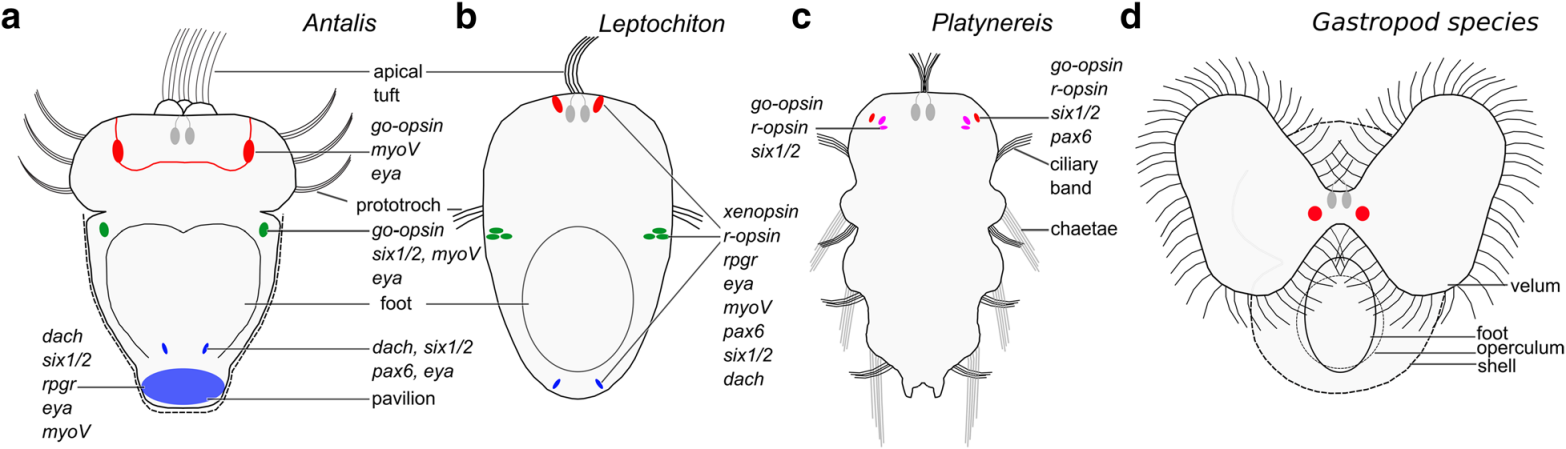
Photoreceptors and gene expression profiles of molluscan and annelid larvae. Ventral views and anterior faces up. The apical (larval) photoreceptors (red) of the trochophore of the scaphopod mollusk *Antalis entalis* (**a**), the trochophore of the polyplacophoran mollusk *Leptochiton asellus* (**b**), the 72 h after fertilization old larva of the polychaete annelid *Platynereis dumerilii* (**c**), and a generalized gastropod veliger larva (**d**) may be homologous based on their cerebral innervation, ontogeny, location close to the apical organ (cells labeled in gray), and their molecular fingerprint. Note that the eyes of gastropod and bivalve veliger larvae have not been characterized based on their gene expression profiles. The scaphopod and polyplacophoran post-trochal photoreceptors (green) are probably homologous since they are located posterior to the prototroch in the mantle and express similar genes. Polyplacophorans possess a pair of posteriormost photoreceptors (blue) in the mantle. The latter may be homologous to scaphopod posterior most expression domains in the pavilion (posterior mantle opening) or the posterior ventral foot based on their location and gene expression profile. The adult eyes of *P. dumerilii* are labeled in pink

Early mid-stage trochophores express *go*-*opsin* in two apical cells located in the lateral episphere adjacent to the trochoblasts, and in two cells of the anterior inner mantle posterior to the prototroch (Figs. 4d–f; 6a; Additional file 1: Figure S2d–f). No shading pigments are associated with these cells. The apical *go*-*opsin*+ cells are flask-shaped, send processes to the apical surface (Fig. 4f), and connect via axons to the neuropilar plexus underlying the apical organ (data not shown). These cells appear to co-express *go*-*opsin*, *eya* and *myoV*, however, we could not unequivocally determine that expression was located within the same cells (Figs. 4g, h; 6a). *Pax6* and *six1/2* are expressed in other flask-shaped apical organ cells, i.e., are not co-expressed with *go*-*opsin*, *eya*, and *myoV* (Figs. 4i; 6a; Additional file 1: Figure S6d–f). *Six1*/*2* and *dach* are expressed in the region of the cerebral ganglia (Fig. 5a, f; Additional file 1: Figures S3d–f, S8d–f). *Go*-*opsin*, *eya*, *six1*/*2*, and *myoV* appear to be co-expressed in two cells of the anterior inner mantle, posterior to the prototroch (Figs. 4d–i; 5a). *Eya* and *six1*/*2* are expressed in the region that connects the hyposphere with the episphere (Figs. 4g, i; 5d, f). The prototroch expresses *rpgr*, while *six1*/*2*, *dach*, *eya*, *myoV* are expressed in several additional regions of the mantle (Figs. 4g–i; 5a, c, e; Additional file 1: Figures S3d–f, S7d–f). *Dach*+, *eya*+, *myoV*+, *pax6*+ and *six1*/*2*+ cells are found in different regions of the foot and form two bilateral co-expression domains in the ventral posterior foot (Figs. 4g–i; 5a, b, d–f; 6a; Additional file 1: Figures S3d–S5d, S6d, e). *Dach*, *six1*/*2*, *rpgr*, *eya*, and *myoV* appear to be co-expressed in a region of the pavilion (Figs. 4g–i; 5a, c–e; 6a; Additional file 1: Figures S3d, S4e, S5e, S7e).

At later stages, the episphere invaginates and accordingly the prototroch is situated more anteriorly [32]. Consequently, both apical *go*-*opsin*+ cells that appear to co-express *eya* and *myoV* are located in the interior of mid-stage trochophores (Fig. 4j–l; Additional file 1: Figures S4h, S5i). Other apical cells also express *pax6* and *six1*/*2*, while *six1*/*2*+ cells are also present in the region of the cerebral and pedal ganglia (Fig. 4m; Additional file 1: Figures S6h, j, S8h, j). *Eya* is expressed in the region connecting the hyposphere and episphere (Fig. 4k). Both post-trochal *go*-*opsin*+ cells are still visible in the mantle and still appear to co-express *eya*, *six1*/*2*, and *myoV* (Fig. 4j–m; Additional file 1: Figures S2g–j). *Dach*, *eya*, *myoV*, *pax6* and *six1*/*2* are expressed in diverse regions of the mantle (Fig. 4j–m; Additional file 1: Figures S3g, S4g–i, S5g–j, S6g–j, S8g–j). The foot houses *dach*+, *myoV*+, and *pax6*+ cells (Additional file 1: Figures S3h, S5i, S6h, i) and portions of the pavilion express *myoV* and *pax6* (Fig. 4l; Additional file 1: Figures S5h, S6h). *Rpgr*+ cells were not detected in the mid-stage trochophore larva (Additional file 1: Figure S7g–j).

## Discussion

### Are scaphopod go-opsins functional?

*Go*-*opsins* are a poorly characterized, but evolutionarily ancient, group of opsin proteins that have been lost in ecdysozoans and many vertebrates [18, 25]. They were first discovered in the ciliary receptors of the distal retina in the mantle eyes of scallops [33]. In annelids, *go*-*opsin1* exhibits a lambda absorption maximum of 488 nm, and these photoreceptors are involved in the phototactic response to light in trochophores and in the mediation of the shadow reflex in adults [34, 35]. Therefore, Go-opsins function in the mediation of light responses in both annelids and mollusks.

In all neuralians studied so far, all opsins are linked via a highly conserved lysine (Schiff base) to a chromophore to form a visual pigment [25]. A covalent interaction between the Schiff base in the seventh transmembrane helix and the retinal chromophore leads to photosensation [36, 37]. In the scaphopod *Antalis entalis*, the predicted Go-opsin amino acid sequence does not contain this lysine (K296, named after the position of the residue in bovine Rhodopsin) (Fig. 2). Absence of the Schiff base is so far only known from fungal, haloarchael, and placozoan opsin-like receptors [25, 38, 39], where it was hypothesized that they may be unable to detect light [25]. However, in vitro experiments indicate that this may not necessarily be the case. Mutation experiments on bovine Rhodopsin revealed that proteins in which K296 had been substituted with another amino acid were constitutively active, i.e., were able to activate signaling via the G-protein transducin in the absence of a chromophore [40]. Further experiments also showed that light-dependent activation for these K296 mutants could be rescued by modification of another residue within the active site to a lysine [26]. In these cases, the protein regains the ability to form a pigment with 11-*cis*-retinal and to activate G-proteins in response to light, although the spectral properties are slightly altered [26, 40]. Prediction of the secondary structure of *A. entalis* Go-opsin revealed that there are no other lysine residues within the retinal-binding pocket (Fig. 3), therefore rescue of responsiveness to light is unlikely to have occurred via this mechanism. Given this, and that the *aen*-*go*-*opsin* sequence contains a domain for G-protein activation (NPIIY motif and tripeptide in Fig. 2), we speculate that the scaphopod Go-opsin may still be functional as a sensory receptor of unknown modality.

### Homology of polyplacophoran and remnant scaphopod apical and post-trochal photoreceptors

*Aen*-*go*-*opsin*+ cells are located in the vicinity of the trochoblasts in the episphere, i.e., a region that may be part of the apical organ (Fig. 4a–c; [32]. Both latter cells are flask shaped, their dendritic processes penetrate the epidermis and their axons run in the direction of the neural plexus underlying the apical organ (data not shown). Therefore, they resemble apical chemoreceptors [32, 41]. The other pair of *aen*-*go*-*opsin*+ cells is present in the inner anterolateral mantle margin posterior to the prototroch in the early mid-stage trochophore (Fig. 4d–f). None of the *aen*-*go*-*opsin*+ cells are accompanied by cells with discrete shading pigments which are required for directional photoreception. Recent studies have demonstrated positive photoresponse behavior without any discrete shading pigment in brachiopods [42], indicating that the opaqueness of the larval body may be used for shielding. Photoreceptors of other mollusks are arranged in a strikingly similar fashion as those of the scaphopod *A. entalis* (Fig. 6a, b). The trochophore of the polyplacophoran *Leptochiton asellus* also possesses a pair of photoreceptor cells in the apical organ or close to it, and the veliger larvae of gastropods and bivalves exhibit cerebrally innervated eyes close to the apical organ (Fig. 6b, d; [17, 18]). The polyplacophoran trochophore additionally possesses a pair of post-trochal larval eyes reminiscent of the scaphopod condition, as well as another pair of photoreceptors at the posteriormost end of the trochophore (Fig. 6b; [17, 18]). Like scaphopod and polyplacophoran trochophore larvae, adult scallops possess photoreceptors within the mantle, albeit within mirror eyes located on the tips of tentacles extending from the middle mantle fold [33]. In contrast to scaphopods (this study), bivalves [33], and gastropods [8], *go*-*opsin* has probably been secondarily lost during evolution in polyplacophorans and cephalopods (*go*-*opsin* appears to be absent in the genome of *Octopus bimaculoides* and no polyplacophoran genome has been published so far). In polyplacophorans, *go*-*opsin* has been functionally replaced by *r*-*opsin* and *xenopsin* within photoreceptors (Fig. 6b; [8, 17, 18, 35]).

### Crucial phototransduction machinery genes are not expressed in scaphopod photoreceptors

To infer whether the scaphopod *go*-*opsin*+ cells possess the genetic inventory for phototransduction, we carried out in situ hybridization experiments on genes involved in phototransduction, ciliary opsin targeting, intracellular R-opsin transport, and eye development as previously reported for the polyplacophoran *L. asellus* [17, 18]. While in *L. asellus* all three groups of photoreceptors co-express *xenopsin*, *r*-*opsin*, *eya*, *dach*, *six1*/*2*, *myoV*, *trypC*, and *rpgr* [17, 18], only few of these genes are potentially co-expressed in the scaphopod *go*-*opsin*+ cells (Fig. 6a, b). *Aen*-*go*-*opsin* appears to be co-expressed with *myoV* and *eya* in the apical cells and *six1*/*2*, *myoV*, and *eya* in the post-trochal cells, but no co-expression was observed with *trpC*, *rpgr*, *pax6*, or *dach* (Fig. 6a). In contrast to apical and post-trochal *go*-*opsin*+ cells which do not express a number of crucial genes implicated in phototransduction or eye development, numerous important phototransduction machinery genes (but not opsins) appear to be co-expressed in few cells of the posterior ventral foot (*dach*, *six1*/*2*, *pax6*, *eya*) and the pavilion (*dach*, *six1*/*2*, *rpgr*, *eya*, *myoV*) (Fig. 6a). This resembles the condition found in the polyplacophoran posterior most photoreceptors that co-express *dach*, *six1*/*2*, *eya*, *pax6*, *rpgr*, and *myoV* in *r*-*opsin*+*/xenopsin*+ cells (Fig. 6b; [17, 18]). While no orthologs of *r*-*opsin* or *c*-*opsin* were found within publicly accessible scaphopod transcriptomic resources, we cannot discount that some of these genes may be co-expressed with *xenopsin*, for which a partial sequence was discovered within the *Antalis entalis* developmental transcriptome. We were unable to amplify this gene from larval cDNA, therefore *Aen*-*xenopsin* may be lowly expressed, or may only be expressed during a very short developmental time frame that has not been considered in our study. The latter condition has been found in cave fish species and their closely related surface-dwelling species that exhibit significantly different *opsin* expression levels depending on the need for short- or long-wavelength sensitivity [43]. Interestingly, only one of the genes identified here (*six1*/*2*) could be found by BLAST within an additional *Antalis entalis* transcriptome dataset [28], (NCBI BioProject PRJNA72139), presumably generated from adult tissue, indicating that expression of the majority of these genes may be restricted to larval stages.

### An evolutionary scenario of molluscan photoreceptor evolution

Based on their ‘cerebral’ expression profile, their topological location, and their distinct cell lineage from other mollusks or annelids, the polyplacophoran post-trochal eyes are argued to have arisen by heterotopic replication from ancestral cerebral eyes under retention of transcriptional activity of genes involved in phototransduction and eye development [17]. Our study highlights a similar location of apical and post-trochal photoreceptors in polyplacophoran and scaphopod trochophores, respectively (Additional file 1: Table S1). The polyplacophoran condition is most similar to the scaphopod early mid-stage trochophore, considered to be the ‘phylotypic stage’ in which staggered Hox gene expression bears most resemblance to the ancestral bilaterian condition (Fig. 6a, b; [44]).

We propose homology of the apical photoreceptor cells of scaphopods, polyplacophorans, and annelids (Fig. 6a–c; [35]). Spatial expression of opsin genes has not been investigated in gastropod and bivalve larvae, however, based on their proximity to the apical organ and their cerebral innervation their larval eyes may be homologous to the scaphopod and polyplacophoran apical photoreceptors (Fig. 6d; Additional file 1: Table S1). Due to a similar location in the trochophore larva and a similar gene expression profile, we propose homology of the post-trochal (mantle) photoreceptors of scaphopods and polyplacophorans. Although adult scallops also express *go*-*opsin* in their photoreceptors within their mantle-based mirror eyes, we hesitate to consider this as support for our hypothesis, since bivalve adult eyes have evolved several times independently and were probably not present in the last common bivalve ancestor (Fig. 1; [33]). Several genes co-expressed in the posterior most polyplacophoran photoreceptors are also expressed in a domain in the posterior scaphopod foot. However, the expression of an opsin in this region could not been proven for scaphopods and therefore the homology of these regions remains unclear.

Given the similarity between polyplacophoran and remnant scaphopod post-trochal photoreceptors, we argue that the evolution of these photoreceptors via heterotopic replication from cerebral eyes may have occurred in the molluscan stem lineage and not only in the polyplacophoran stem lineage. If correct, larval post-trochal/mantle edge-associated eyes could therefore be considered a molluscan (not a polyplacophoran) synapomorphy, with loss of post-trochal (mantle margin) photoreceptors in other mollusks. Alternatively, the evolution of post-trochal eyes may have occurred via the gradual co-option of photoreceptor gene expression to the post-trochal region in both polyplacophorans and scaphopods. Although additional data are needed, we consider the first evolutionary scenario more parsimonious as it could have occurred via the change of expression of a single regulatory gene, while stepwise co-option of single genes of a gene regulatory network requires several evolutionary events.

## Conclusions

Our results indicate that the scaphopod *go*-*opsin*+ cells represent former photoreceptors which have probably evolved into receptors of another sensory modality. This degeneration can be seen in the light-insensitive Go-opsin, the loss of photoreceptor shielding pigments, and the loss of expression of several genes involved in phototransduction and eye development. The similar topographical constellation of remnant photoreceptor cells with functional photoreceptors in polyplacophorans suggests that the last common molluscan ancestor also possessed apical, post-trochal, and probably posterior photoreceptors, which represent previously unrecognized molluscan synapomorphies.

## Methods

### Ethics, collection and culture of animals

Adults of the scaphopod *Antalis entalis* Jeffreys 1869 were collected from approximately 25 m depth by the staff of the research vessel Neomys off the coast of Roscoff (France) in the summer of 2014 and 2017. Animals spawned and developmental stages were reared at 18–20 °C as described previously [16].

### RNA extraction and fixation of animals for in situ hybridization experiments

Several hundred individuals of early cleavage stages to settled metamorphosed individuals were investigated. All developmental stages were carefully anesthetized in 7.14% MgCl_2_ before fixation for in situ hybridization experiments as previously described [16].

### Alignment, phylogenetic analysis and secondary structure prediction

Candidate genes were identified by BLAST searches against the published transcriptomes of the scaphopod *A. entalis* ([16, 27]; NCBI bioproject PRJNA357466; assembly available at https://zoology.univie.ac.at/open-data/) were derived from pooled RNA from several hundred specimens of early embryos to postmetamorphic individuals. Phylogenetic analyses were performed for each of the predicted protein sequences building upon the analyses of Vöcking et al. [17, 18] and Ramirez et al. [8]. Sequences were aligned and manually edited within AliView [45], and maximum likelihood phylogenetic analyses were performed using RAxML 8.2.11 [46] with automatic model selection, gamma model of rate heterogeneity, and 100 bootstrap replicates. Phylogenetic trees were visualized and edited in FigTree [47]. Protein secondary structure and ligand binding prediction were performed using I-TASSER [48]. Resulting PDB models were viewed, annotated, and aligned with bovine rhodopsin (PDB ID code 1U19) in UCSF Chimera [49].

### Molecular isolation of RNA transcripts

A first-strand cDNA Synthesis Kit for rt-PCR (Roche Diagnostics GmbH, Mannheim, Germany) was used for first-strand cDNA synthesis of the RNA pooled from different developmental stages of *A. entalis* [16]. Identified gene sequences were used to design gene-specific primers (see Additional file 1) and PCR products were size fractioned by gel electrophoresis, gel bands of the expected lengths were excised and cleaned up using a QIAquick Gel Extraction Kit (QIAgen, Hilden, Germany). Cleaned-up products were cloned by insertion into pGEM-T Easy Vectors (Promega, Mannheim, Germany), as per the manufacturer’s protocol. Plasmid minipreps were grown overnight, cleaned up with the QIAprep Spin Miniprep Kit (QIAgen), and sent for sequencing to confirm identity.

### Probe synthesis and whole-mount in situ hybridization

Riboprobe templates were amplified via standard PCR from miniprep plasmids using M13 forward and reverse primers as described previously [16]. In vitro transcription reactions were performed with these templates, digoxigenin-UTP (DIG RNA Labeling Kit, Roche Diagnostics), and SP6/T7 polymerase (Roche Diagnostics GmbH) for the synthesis of antisense riboprobes, according to the manufacturer’s instructions. For whole-mount in situ hybridization experiments, specimens were rehydrated into PBT (phosphate buffered saline + 0.1% Tween-20) and treated with Proteinase-K at 37 °C for 10 min (30 µg/mL in PBT). Specimens were pre-hybridized in hybridization buffer for 4–10 h at 58 °C (see [16] for details). Hybridization was performed at the same temperature with probe concentrations ranging between 1 and 2 μg/mL for 21–24 h. A DIG-labeled AP antibody was used at a dilution of 1:2500 in blocking solution at 4 °C overnight. Color development in the NBT/BCIP/alkaline phosphatase buffer solution took 6–24 h at 4 °C. Some specimens were counterstained with DAPI to visualize cell nuclei (Sigma-Aldrich, St. Louis, MO, USA). A minimum of 30 individuals per stage were investigated. The majority of whole-mount preparations were cleared in a solution of 2,2′thiodiethanol (Sigma-Aldrich), mounted on objective slides and analyzed. Preparations were documented with an Olympus BX53 Microscope (Olympus, Hamburg, Germany). In addition, developmental stages were scanned with a Leica confocal SP5 II microscope (Leica Microsystems, Wetzlar, Germany) using bright-field, autofluorescence, and reflection mode scans to document the precise cellular location of transcripts [50]. If necessary, images were processed with Adobe Photoshop 9.0.2 software (San Jose, CA, USA) to adjust for contrast and brightness. Sketch drawings were created with Adobe Illustrator CC 2015.1.0 (Adobe Systems, Inc., San Jose, CA; USA). The absence of staining caused by endogenous alkaline phosphatases has previously been demonstrated, and expression patterns shown within this study were distinct from those of off-target controls ([16, 44]; Additional file 1: Figure S9)

## Supplementary information

**Additional file 1: Figures S1a–h.** Phylogenetic analyses. **Figures S2–S9.** Additional gene expression patterns. **Table S1.** Primer and Go-opsin sequences.

## Data Availability

All sequences analyzed in this study have been published on publicly accessible websites.

## Abbreviations

*Aen*: *Antalis entalis*
BCIP: 5-brom-4-chlor-3-indoxylphosphat
BLAST: Basic local alignment search tool
C-opsin: Ciliary opsin
cDNA: Complementary deoxyribonucleic acid
cg: Cerebral ganglia
DIG: Digoxigenin
*eya*: *eyes absent*
f: Foot
*dach*: *dachshund*
NBT: Nitro blue tetrazolium
NCBI: National center for biotechnology information
*myoV*: *myosinV*
*pax6*: *paired box protein 6*
PBT: Phosphate-buffered saline with Triton X-100
pcr: Polymerase chain reaction
pv: Pavilion
pt: Prototroch
RNA: Ribonucleic acid
r-opsin: Rhabdomeric opsin
*rpgr*: *retinitis pigmentosa GTPase regulator*
*six1*/*2*: *sine oculis homeobox gene 1*/*2*
*trpC*: *transient receptor potential cation channel*
