# Supplementary material for: Remnants of ancestral larval eyes in an eyeless mollusk? Molecular characterization of photoreceptors in the scaphopod Antalis entalis
Source: EvoDevo. 2019 Oct 19;10:25. doi: 10.1186/s13227-019-0140-7 (PMC6800502; doi:10.1186/s13227-019-0140-7)
Supplement: Supplementary file 1 — Additional file 1: Figures S1a–h. Phylogenetic analyses. Figures S2–S9. Additional gene expression patterns. Table S1. Primer and Go-opsin sequences. [file 13227_2019_140_MOESM1_ESM.pdf]

Additional file 1. Phylogenetic analyses (Figures S1a-h), additional gene expression patterns (Figures S2-9), Table S1, primer and Go-opsin sequences.

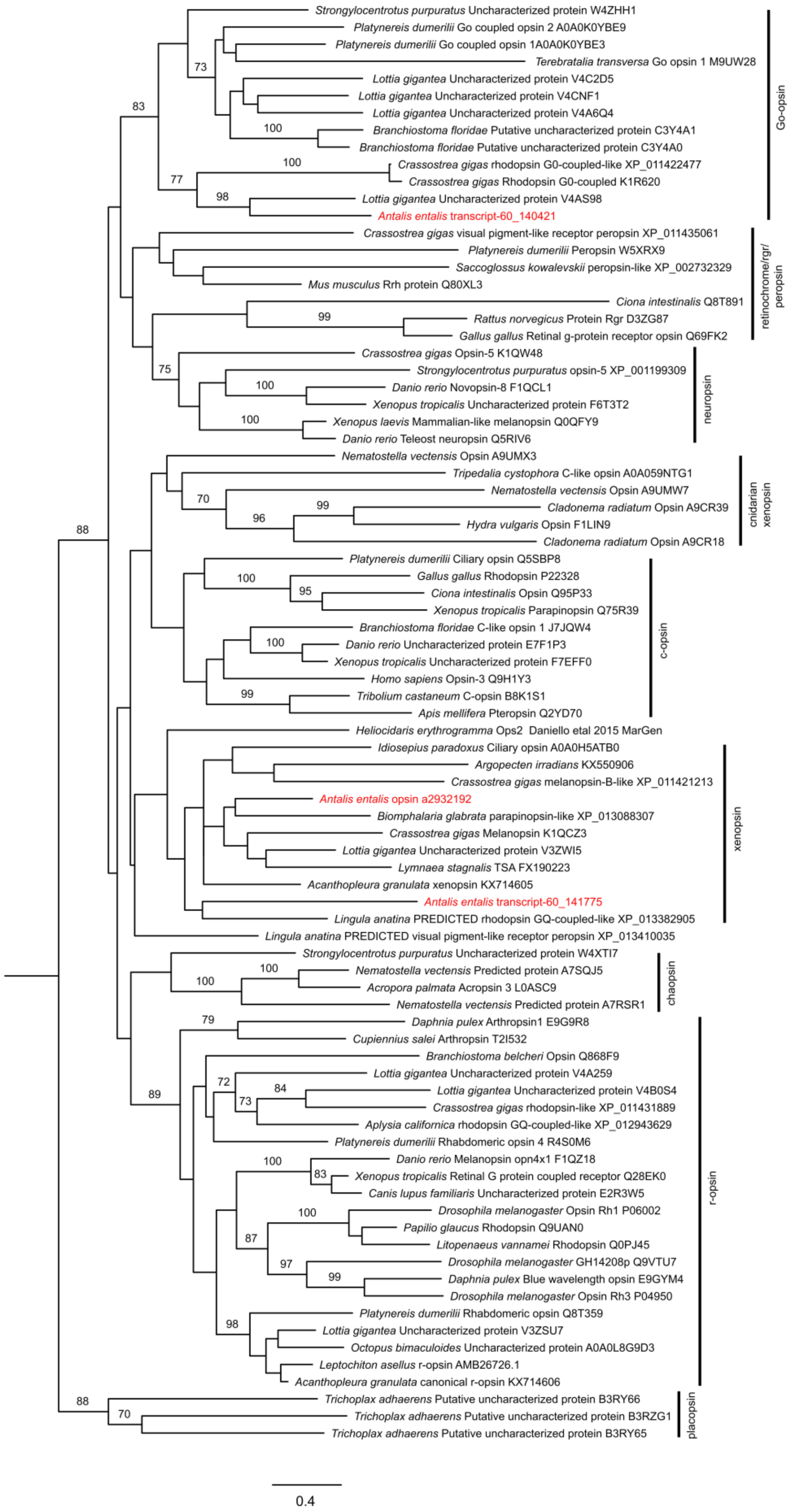

Strongylocentrotus purpuratus Uncharacterized protein W4ZHH1
Platynereis dumerilii Go coupled opsin 2 A0A0K0YBE9
Platynereis dumerilii Go coupled opsin 1A0A0K0YBE3
Terebratalia transversa Go opsin 1 M9UW28
Lottia gigantea Uncharacterized protein V4C2D5
Lottia gigantea Uncharacterized protein V4CNF1
Lottia gigantea Uncharacterized protein V4A6Q4
Branchiostoma floridae Putative uncharacterized protein C3Y4A1
Branchiostoma floridae Putative uncharacterized protein C3Y4A0
Crassostrea gigas rhodopsin G0-coupled-like XP_011422477
Crassostrea gigas Rhodopsin G0-coupled K1R620
Lottia gigantea Uncharacterized protein V4AS98
Antalis entalis transcript-60_140421
Go-opsin
Crassostrea gigas visual pigment-like receptor peropsin XP_011435061
Platynereis dumerilii Peropsin W5XRX9
Saccoglossus kowalevskii peropsin-like XP_002732329
Mus musculus Rrh protein Q80XL3
Ciona intestinalis Q8T891
Rattus norvegicus Protein Rgr D3ZG87
Gallus gallus Retinal g-protein receptor opsin Q69FK2
retinochrome/rgr/ peropsin
Crassostrea gigas Opsin-5 K1QW48
Strongylocentrotus purpuratus opsin-5 XP_001199309
Danio rerio Novopsin-8 F1QCL1
Xenopus tropicalis Uncharacterized protein F6T3T2
Xenopus laevis Mammalian-like melanopsin Q0QFY9
Danio rerio Teleost neuropsin Q5RIV6
neuropsin
Nematostella vectensis Opsin A9UMX3
Tripedalia cystophora C-like opsin A0A059NTG1
Nematostella vectensis Opsin A9UMW7
Cladonema radiatum Opsin A9CR39
Hydra vulgaris Opsin F1LIN9
Cladonema radiatum Opsin A9CR18
cnidarian xenopsin
Platynereis dumerilii Ciliary opsin Q5SBP8
Gallus gallus Rhodopsin P22328
Ciona intestinalis Opsin Q95P33
Xenopus tropicalis Parapinopsin Q75R39
Branchiostoma floridae C-like opsin 1 J7JQW4
Danio rerio Uncharacterized protein E7F1P3
Xenopus tropicalis Uncharacterized protein F7EFF0
Homo sapiens Opsin-3 Q9H1Y3
Tribolium castaneum C-opsin B8K1S1
Apis mellifera Pteropsin Q2YD70
c-opsin
Heliocidaris erythrogramma Ops2 Daniello etal 2015 MarGen
Idiosepius paradoxus Ciliary opsin A0A0H5ATB0
Argopecten irradians KX550906
Crassostrea gigas melanopsin-B-like XP_011421213
Antalis entalis opsin a2932192
Biomphalaria glabrata parapinopsin-like XP_013088307
Crassostrea gigas Melanopsin K1QCZ3
Lottia gigantea Uncharacterized protein V3ZWI5
Lymnaea stagnalis TSA FX190223
Acanthopleura granulata xenopsin KX714605
Antalis entalis transcript-60_141775
Lingula anatina PREDICTED rhodopsin GQ-coupled-like XP_013382905
xenopsin
Lingula anatina PREDICTED visual pigment-like receptor peropsin XP_013410035
Strongylocentrotus purpuratus Uncharacterized protein W4XTI7
Nematostella vectensis Predicted protein A7SQJ5
Acropora palmata Acropsin 3 L0ASC9
Nematostella vectensis Predicted protein A7RSR1
chaopsin
Daphnia pulex Arthropsin1 E9G9R8
Cupiennius salei Arthropsin T2I532
Branchiostoma belcheri Opsin Q868F9
Lottia gigantea Uncharacterized protein V4A259
Lottia gigantea Uncharacterized protein V4B0S4
Crassostrea gigas rhodopsin-like XP_011431889
Aplysia californica rhodopsin GQ-coupled-like XP_012943629
Platynereis dumerilii Rhabdomeric opsin 4 R4S0M6
Danio rerio Melanopsin opn4x1 F1QZ18
Xenopus tropicalis Retinal G protein coupled receptor Q28EK0
Canis lupus familiaris Uncharacterized protein E2R3W5
Drosophila melanogaster Opsin Rh1 P06002
Papilio glaucus Rhodopsin Q9UAN0
Litopenaeus vannamei Rhodopsin Q0PJ45
Drosophila melanogaster GH14208p Q9VTU7
Daphnia pulex Blue wavelength opsin E9GYM4
Drosophila melanogaster Opsin Rh3 P04950
Platynereis dumerilii Rhabdomeric opsin Q8T359
Lottia gigantea Uncharacterized protein V3ZSU7
Octopus bimaculoides Uncharacterized protein A0A0L8G9D3
Leptochiton asellus r-opsin AMB26726.1
Acanthopleura granulata canonical r-opsin KX714606
r-opsin
Trichoplax adhaerens Putative uncharacterized protein B3RY66
Trichoplax adhaerens Putative uncharacterized protein B3RZG1
Trichoplax adhaerens Putative uncharacterized protein B3RY65
placopsin
0.4

Figure S1a. Phylogenetic analyses on metazoan opsins.

Maximum likelihood phylogenetic analysis of the *Antalis entalis* opsin proteins (red). NCBI accession numbers are provided for each sequence (accession number for *aen-xenopsin*: MK934770). Tree is based upon that presented in Ramirez et al. 2016. Percent bootstrap values over 70 are displayed, and the scale bar indicates the branch length for 0.4 amino acid substitutions. *Trichoplax* opsin proteins (placopsins) are included as an outgroup.

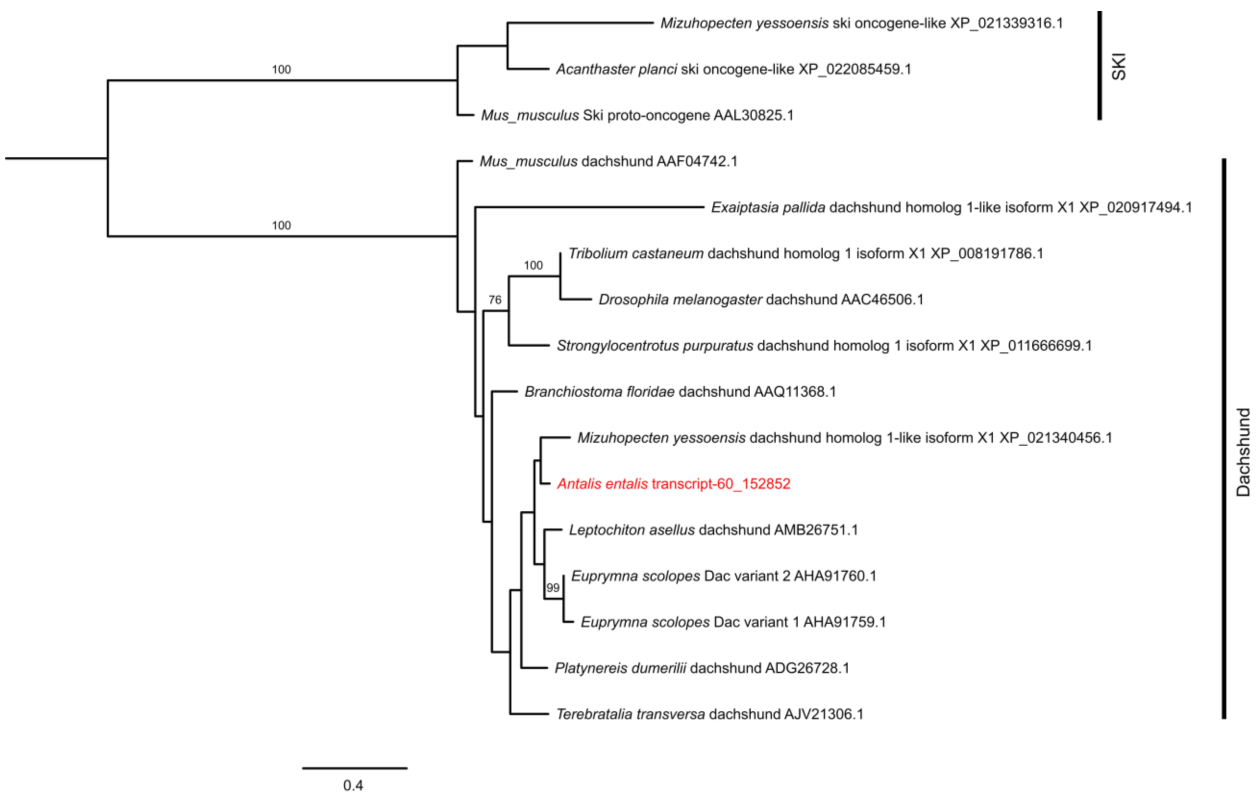

Figure S1b. Maximum likelihood phylogenetic analysis of the *Antalis entalis* Dachshund protein (red). NCBI accession numbers are provided for each sequence. Percent bootstrap values over 70 are displayed, and the scale bar indicates the branch length for 0.4 amino acid substitutions. Related ski proteins are included as an outgroup.

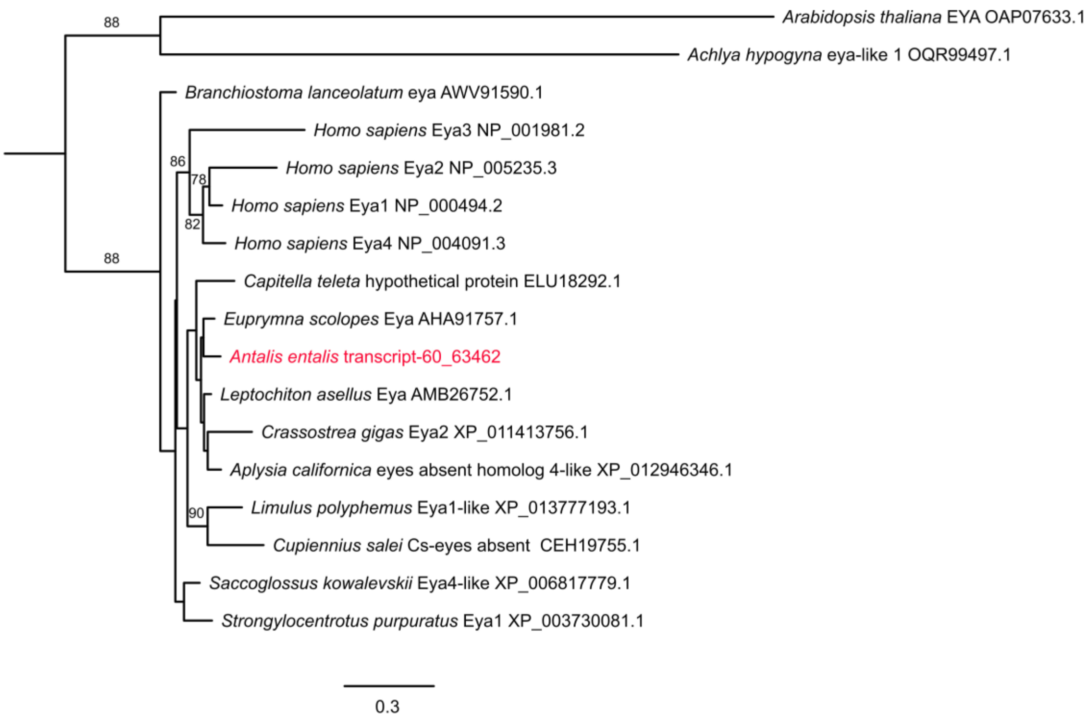

Figure S1c. Maximum likelihood phylogenetic analysis of the *Antalis entalis* Eya protein (red). NCBI accession numbers are provided for each sequence. Percent bootstrap values over 70 are displayed, and the scale bar indicates the branch length for 0.3 amino acid substitutions. Non-metazoan Eya sequences are included as an outgroup.

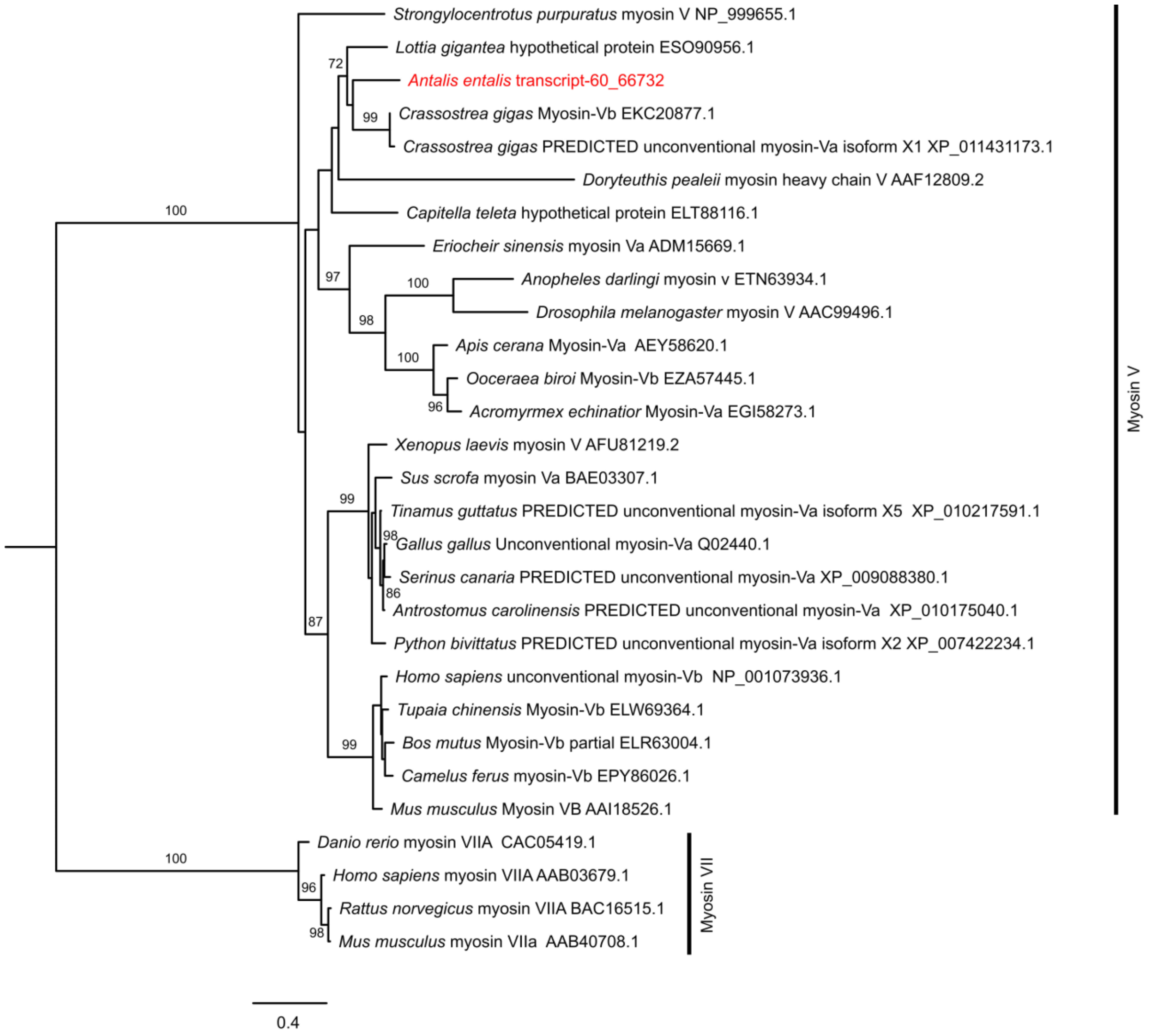

Figure S1d. Maximum likelihood phylogenetic analysis of the *Antalis entalis* Myosin V protein (red). NCBI accession numbers are provided for each sequence. Tree is based upon that presented in Vöcking et al. (2015). Percent bootstrap values over 70 are displayed, and the scale bar indicates the branch length for 0.4 amino acid substitutions. Myosin VII proteins are included as an outgroup.

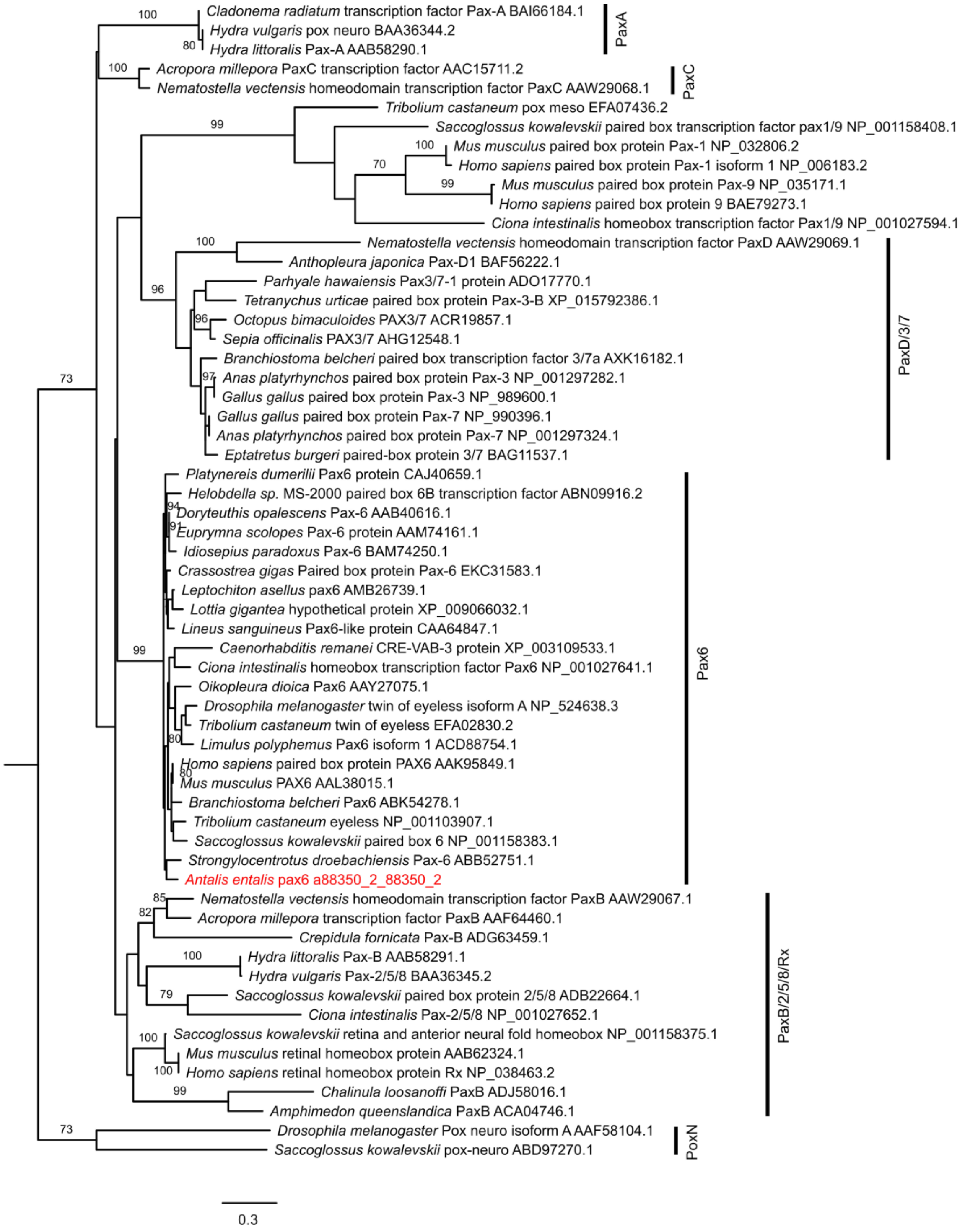
Cladonema radiatum transcription factor Pax-A BAI66184.1
Hydra vulgaris pox neuro BAA36344.2
Hydra littoralis Pax-A AAB58290.1
Acropora millepora PaxC transcription factor AAC15711.2
Nematostella vectensis homeodomain transcription factor PaxC AAW29068.1
Tribolium castaneum pox meso EFA07436.2
Saccoglossus kowalevskii paired box transcription factor pax1/9 NP_001158408.1
Mus musculus paired box protein Pax-1 NP_032806.2
Homo sapiens paired box protein Pax-1 isoform 1 NP_006183.2
Mus musculus paired box protein Pax-9 NP_035171.1
Homo sapiens paired box protein 9 BAE79273.1
Ciona intestinalis homeobox transcription factor Pax1/9 NP_001027594.1
Nematostella vectensis homeodomain transcription factor PaxD AAW29069.1
Anthopleura japonica Pax-D1 BAF56222.1
Parhyale hawaiensis Pax3/7-1 protein ADO17770.1
Tetranychus urticae paired box protein Pax-3-B XP_015792386.1
Octopus bimaculoides PAX3/7 ACR19857.1
Sepia officinalis PAX3/7 AHG12548.1
Branchiostoma belcheri paired box transcription factor 3/7a AXK16182.1
Anas platyrhynchos paired box protein Pax-3 NP_001297282.1
Gallus gallus paired box protein Pax-3 NP_989600.1
Gallus gallus paired box protein Pax-7 NP_990396.1
Anas platyrhynchos paired box protein Pax-7 NP_001297324.1
Eptatretus burgeri paired-box protein 3/7 BAG11537.1
Platynereis dumerilii Pax6 protein CAJ40659.1
Helobdella sp. MS-2000 paired box 6B transcription factor ABN09916.2
Doryteuthis opalescens Pax-6 AAB40616.1
Euprymna scolopes Pax-6 protein AAM74161.1
Idiosepius paradoxus Pax-6 BAM74250.1
Crassostrea gigas Paired box protein Pax-6 EKC31583.1
Leptochiton asellus pax6 AMB26739.1
Lottia gigantea hypothetical protein XP_009066032.1
Lineus sanguineus Pax6-like protein CAA64847.1
Caenorhabditis remanei CRE-VAB-3 protein XP_003109533.1
Ciona intestinalis homeobox transcription factor Pax6 NP_001027641.1
Oikopleura dioica Pax6 AAY27075.1
Drosophila melanogaster twin of eyeless isoform A NP_524638.3
Tribolium castaneum twin of eyeless EFA02830.2
Limulus polyphemus Pax6 isoform 1 ACD88754.1
Homo sapiens paired box protein PAX6 AAK95849.1
Mus musculus PAX6 AAL38015.1
Branchiostoma belcheri Pax6 ABK54278.1
Tribolium castaneum eyeless NP_001103907.1
Saccoglossus kowalevskii paired box 6 NP_001158383.1
Strongylocentrotus droebachiensis Pax-6 ABB52751.1
Antalis entalis pax6 a88350_2_88350_2
Nematostella vectensis homeodomain transcription factor PaxB AAW29067.1
Acropora millepora transcription factor PaxB AAF64460.1
Crepidula fornicata Pax-B ADG63459.1
Hydra littoralis Pax-B AAB58291.1
Hydra vulgaris Pax-2/5/8 BAA36345.2
Saccoglossus kowalevskii paired box protein 2/5/8 ADB22664.1
Ciona intestinalis Pax-2/5/8 NP_001027652.1
Saccoglossus kowalevskii retina and anterior neural fold homeobox NP_001158375.1
Mus musculus retinal homeobox protein AAB62324.1
Homo sapiens retinal homeobox protein Rx NP_038463.2
Chalinula loosanoffi PaxB ADJ58016.1
Amphimedon queenslandica PaxB ACA04746.1
Drosophila melanogaster Pox neuro isoform A AAF58104.1
Saccoglossus kowalevskii pox-neuro ABD97270.1
PaxA
PaxC
Pax1/9
PaxD/3/7
Pax6
PaxB/2/5/8/Rx
PoxN
100
80
100
99
100
70
99
100
96
96
97
73
94
91
99
80
80
85
82
100
79
100
100
99
73
0.3

Figure S1e. Maximum likelihood phylogenetic analysis of the *Antalis entalis* Pax6 protein (red). NCBI accession numbers are provided for each sequence (accession number for *aen-pax6*: MK934769). Tree is based upon that presented in Vöcking et al. (2015). Percent bootstrap values over 70 are displayed, and the scale bar indicates the branch length for 0.3 amino acid substitutions. Pox neuro proteins are included as an outgroup.

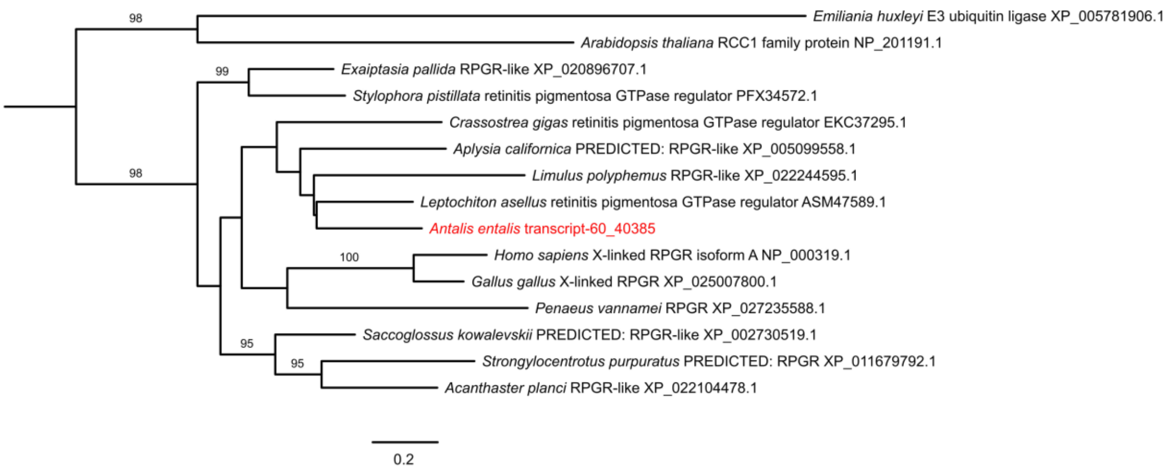

Figure 1f. Maximum likelihood phylogenetic analysis of the *Antalis entalis* Rpgr protein (red). NCBI accession numbers are provided for each sequence. Percent bootstrap values over 70 are displayed, and the scale bar indicates the branch length for 0.2 amino acid substitutions. Related RCC1-containing proteins are included as an outgroup.

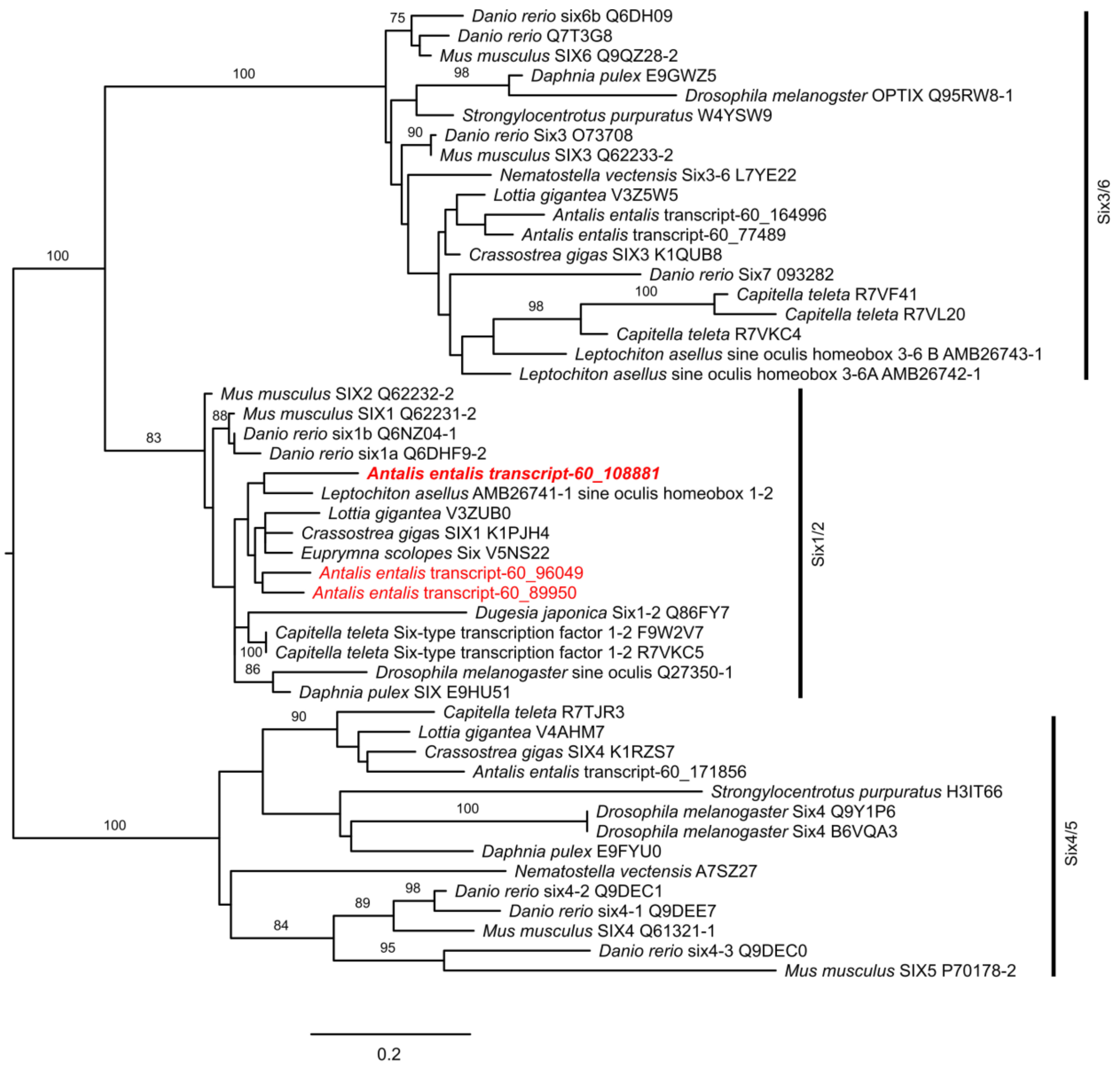

Figure S1g. Maximum likelihood phylogenetic analysis of the *Antalis entalis* Six1/2 proteins (red). Three transcripts encode proteins that fall within the Six1/2 clade, the sequence with the highest similarity to *L. asellus* Six1/2 is bold. NCBI or UniProt accession numbers are provided for all sequences. Tree is based upon that presented in Vöcking et al. (2015). Percent bootstrap values over 70 are displayed, and the scale bar indicates the branch length for 0.2 amino acid substitutions. Midpoint rooting.

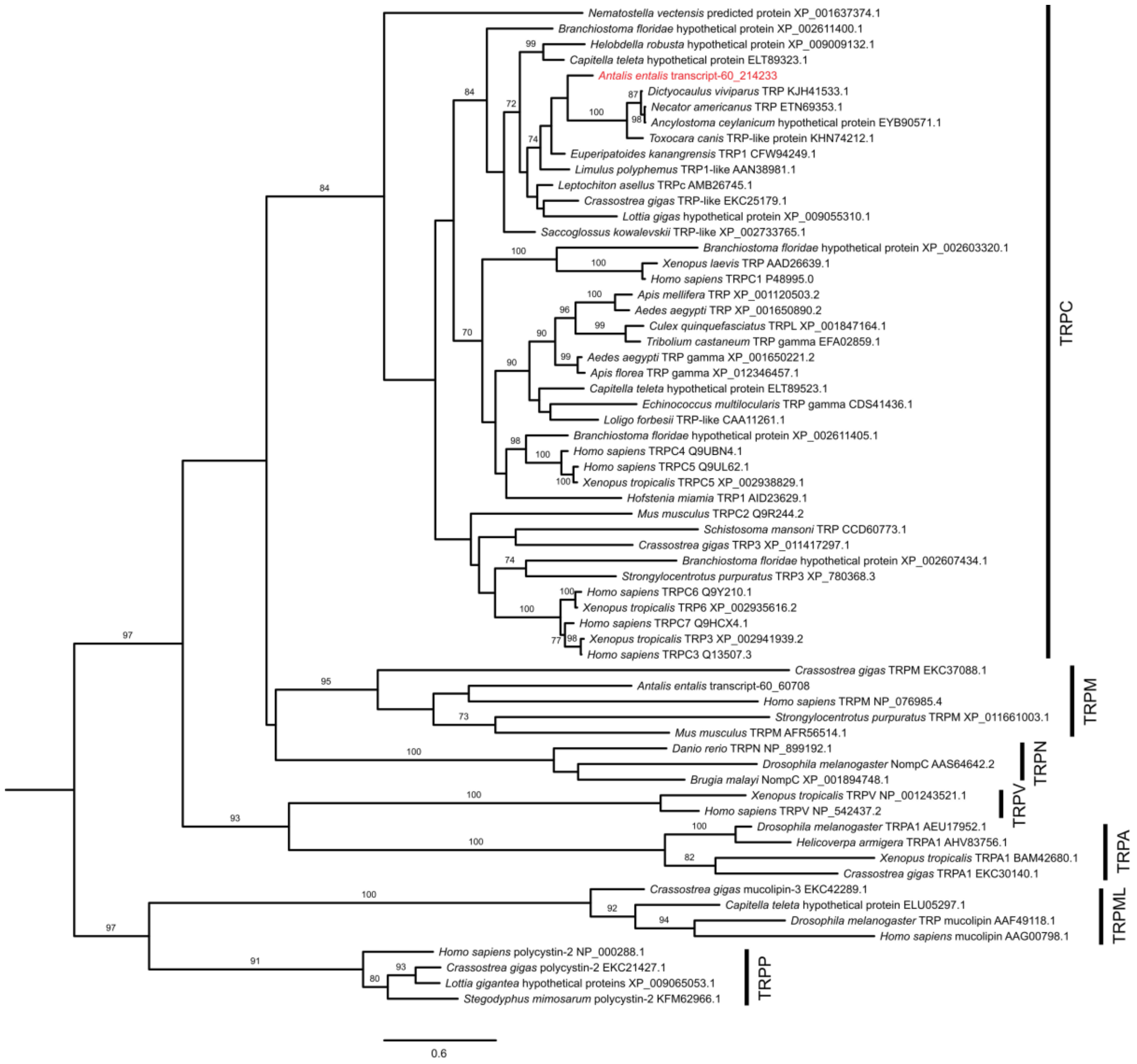

Figure S1h. Maximum likelihood phylogenetic analysis of the *Antalis entalis* TRPC protein (red). NCBI accession numbers are provided for each sequence. Tree is based upon that represented in Vöcking et al. (2015). Percent bootstrap values over 70 are displayed, and the scale care indicates the branch length for 0.6 amino acid substitutions. Midpoint rooting.

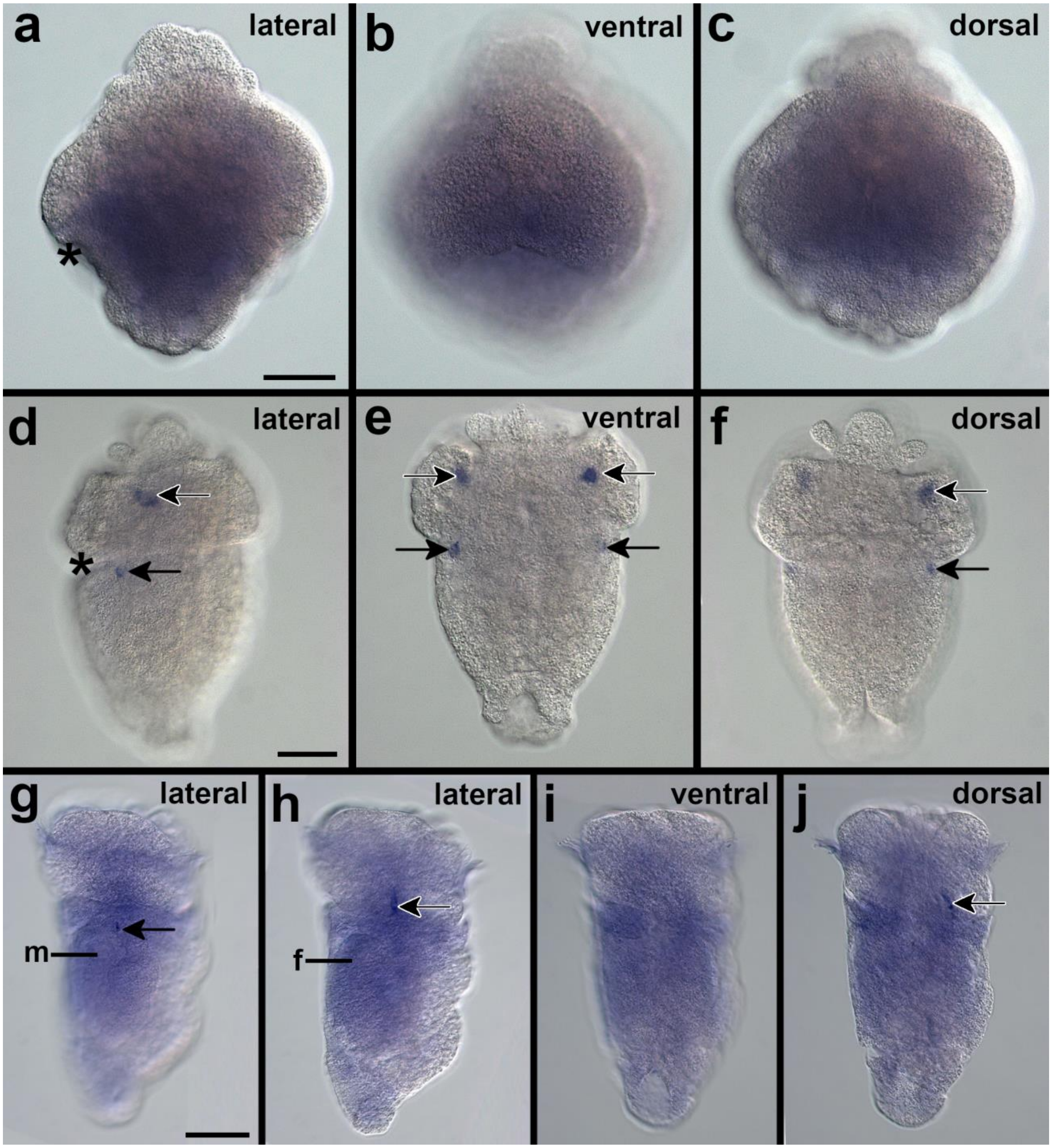

Figure S2. ***Go-opsin*** **expression during the development of the scaphopod** ***Antalis entalis*****.** Anterior faces up in all panels and dorsal faces to the right in lateral views. Asterisk labels the mouth. (**a-c**) Early trochophore larvae express *go-opsin* in the region around the mouth. **(d-f)** Early mid-stage trochophores express *go-opsin* in two cells adjacent to the apical organ (white-lined arrows) and two cells in the anterior inner mantle (black arrows). **(g-j)** Latero-distal view

(g), latero-proximal view **(h)**, ventral view **(i)**, and dorsal view **(j)** of mid-stage trochophore larvae that express *go-opsin* in cells migrating to the posterior pole of the trochophore larvae. Both other cells embedded in the antero-lateral mantle margin do still exist (black arrow). Abbreviations: f foot; m mantle, pt prototroch. Scale bars: 50 µm.

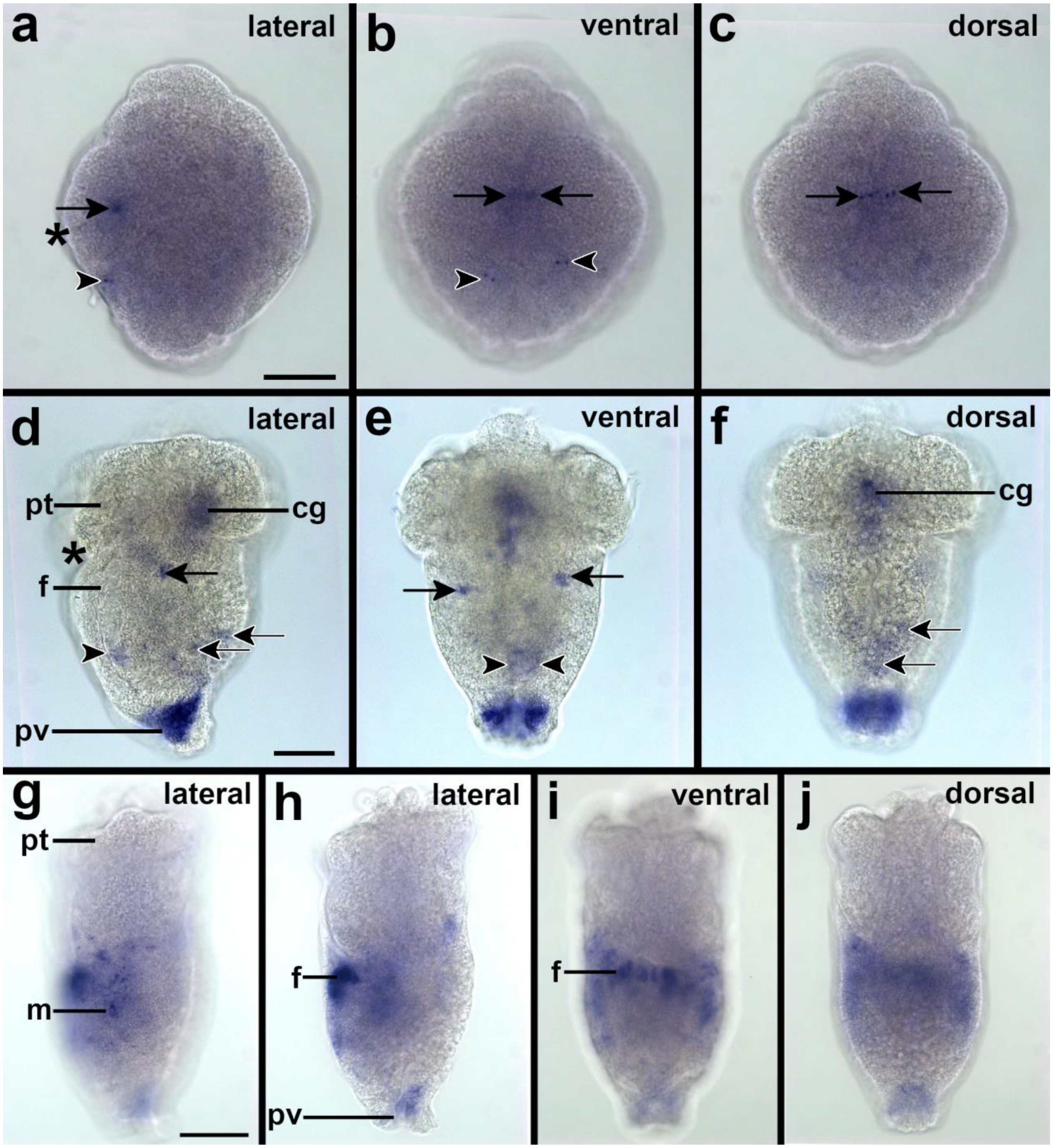

Figure S3. ***Dach*** **expression during the development of the scaphopod** ***Antalis entalis*****.** Anterior faces up in all panels and dorsal faces to the right in lateral views. Asterisk labels the mouth. (**a-c)** Early trochophore larvae express *dach* in each two bilateral cells (white-lined arrowheads) embedded in the epidermis posterior to the mouth and in two cells close to the foregut (black arrows). **(d-f)** Early mid-stage trochophores express *dach* in the region of the cerebral ganglia, the pavilion, the antero-lateral and posterior foot, the posterior dorsal mantle region, and the lateral foot. **(g-j)** Latero-distal view **(g)**, latero-proximal view **(h)**, ventral view **(i)**, and dorsal view **(j)** of mid-stage trochophore larvae that express *dach* in cells of the mantle, the foot, and pavilion. Abbreviations: cg cerebral ganglion, f foot, m mantle, pt prototroch, pv pavilion. Scale bars: 50 µm.

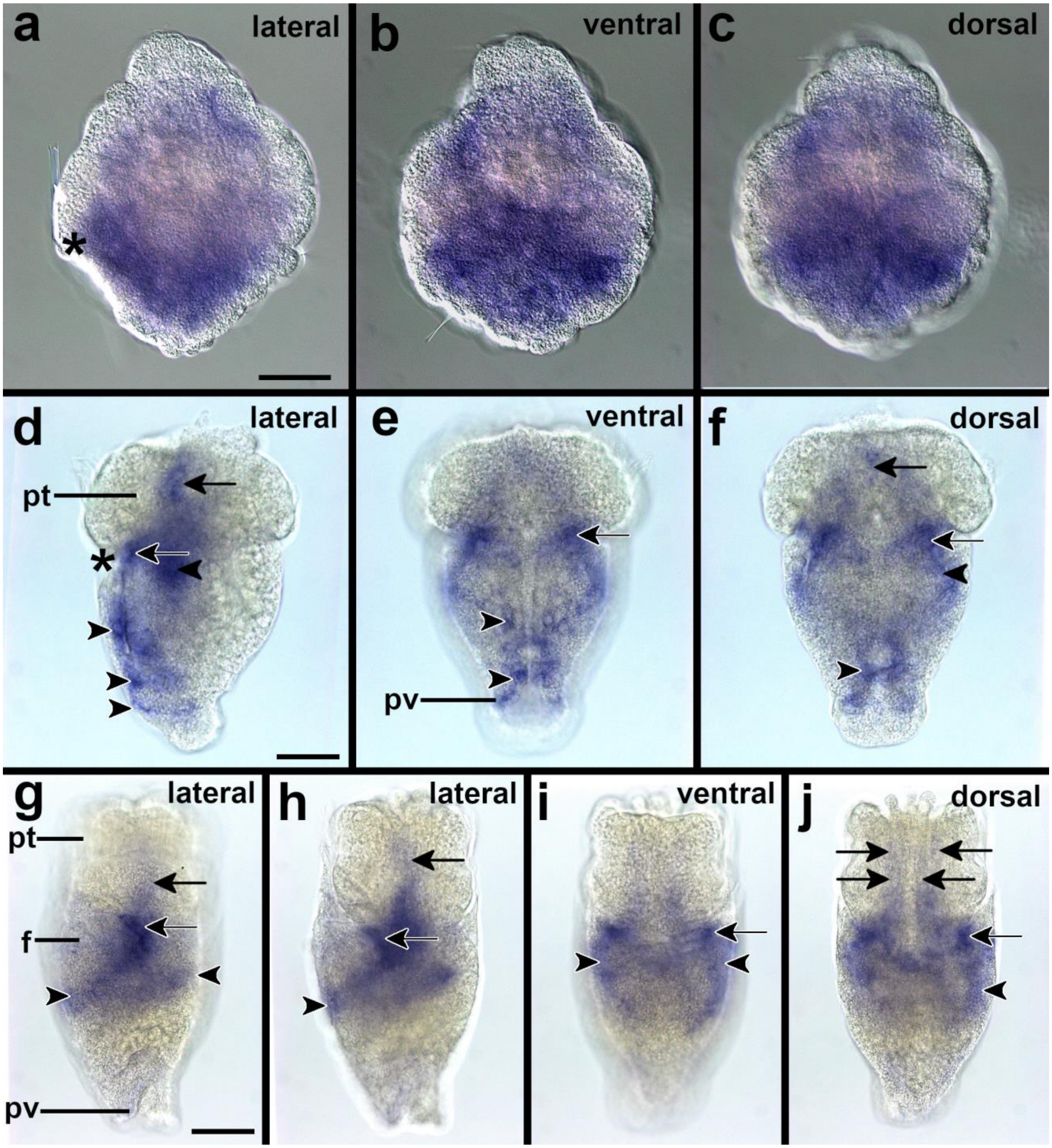

Figure S4. *Eya* expression during the development of the scaphopod *Antalis entalis*. Anterior faces up in all panels and dorsal faces to the right in lateral views. Asterisk labels the mouth. (**a-c**) Early trochophore larvae express *eya* globally in the interior of the larva. **(d-f)** Early mid-stage trochophores express *eya* in cells of the apical organ (arrowheads), in the region connecting the hyposphere with the invaginating episphere and the prototroch (white-lined arrows), and in

individual cells of the ventral mantle (white-lined arrowheads). **(g-j)** Latero-distal view **(g)**, latero-proximal view **(h)**, ventral view **(i)**, and dorsal view **(j)** of mid-stage trochophore larvae that express *eya* in apical organ cells (arrows), the mantle (white-lined arrowheads), the region connecting trunk with episphere and prototroch (white-lined arrows), and the pavilion. Abbreviations: f foot, pt prototroch, pv pavilion. Scale bars: 50 µm.

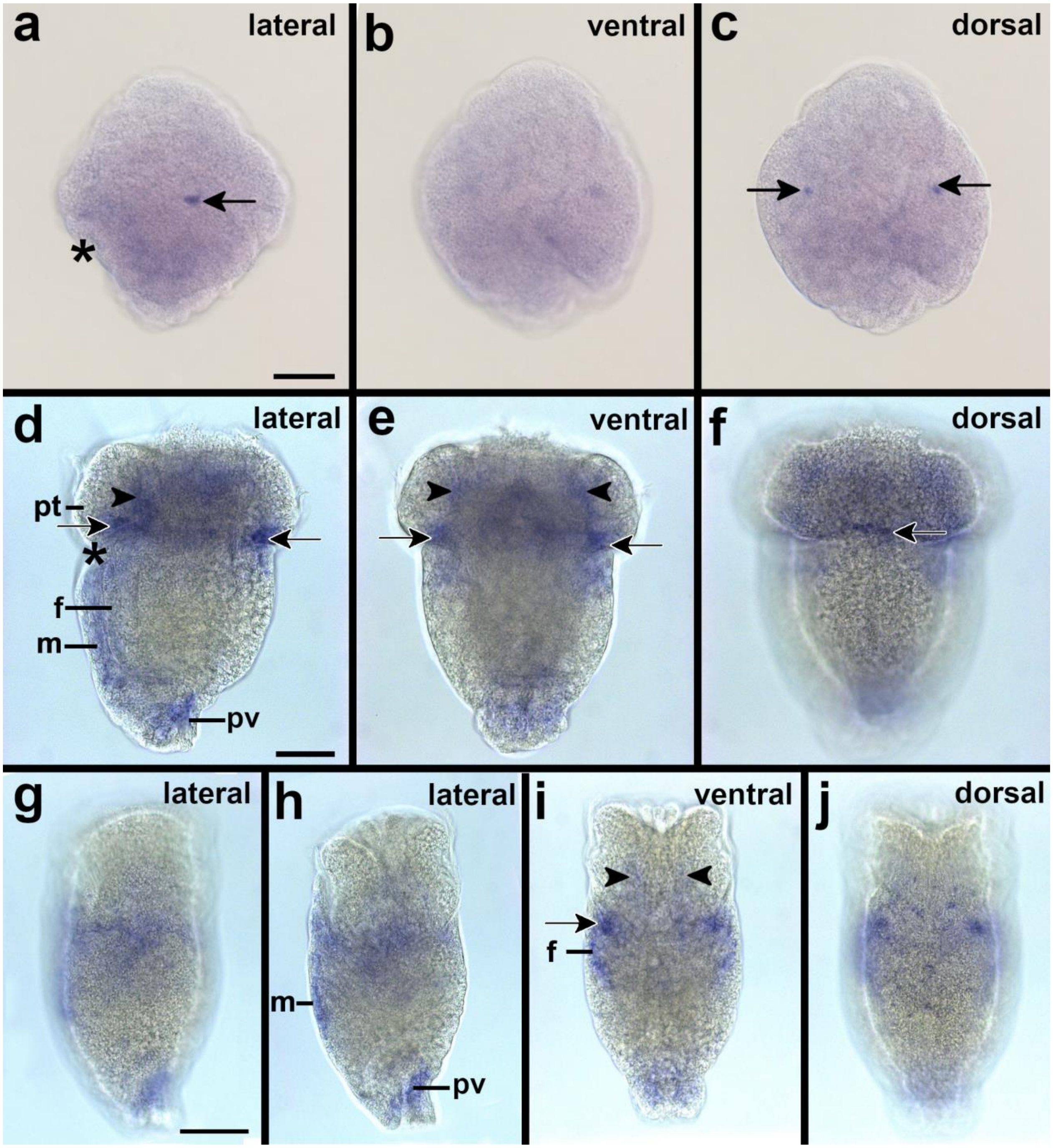

Figure S5. ***MyoV* expression during the development of the scaphopod *Antalis entalis*.** Anterior faces up in all panels and dorsal faces to the right in lateral views. Asterisk labels the mouth. (**a-c)** Early trochophore larvae express *myoV*+ in two cells that are located on the level of the prototroch inside the early trochophore larva. **(d-f)** In early mid-stage trochophores *myoV*+ cells line the mantle and the foot and are present in the pavilion. *MyoV* is strongly expressed in the dorsal and ventral anterior mantle margins (white-lined arrows). Several cells of the episphere including the apical organ express *myoV*. *MyoV*+ cells are present in the apical organ (black arrowheads). **(g-j)** Latero-distal view **(g)**, latero-proximal view **(h)**, ventral view **(i)**, and dorsal view **(j)** of mid-stage trochophore larvae that expresses *myoV*+ in cells in similar location as early mid-stage trochophores, however, apical *myoV*+ cells migrate into the interior of the trochophore larvae (s. above). Abbreviations: f foot, m mantle, pt prototroch, pv pavilion. Scale bars: 50 µm.

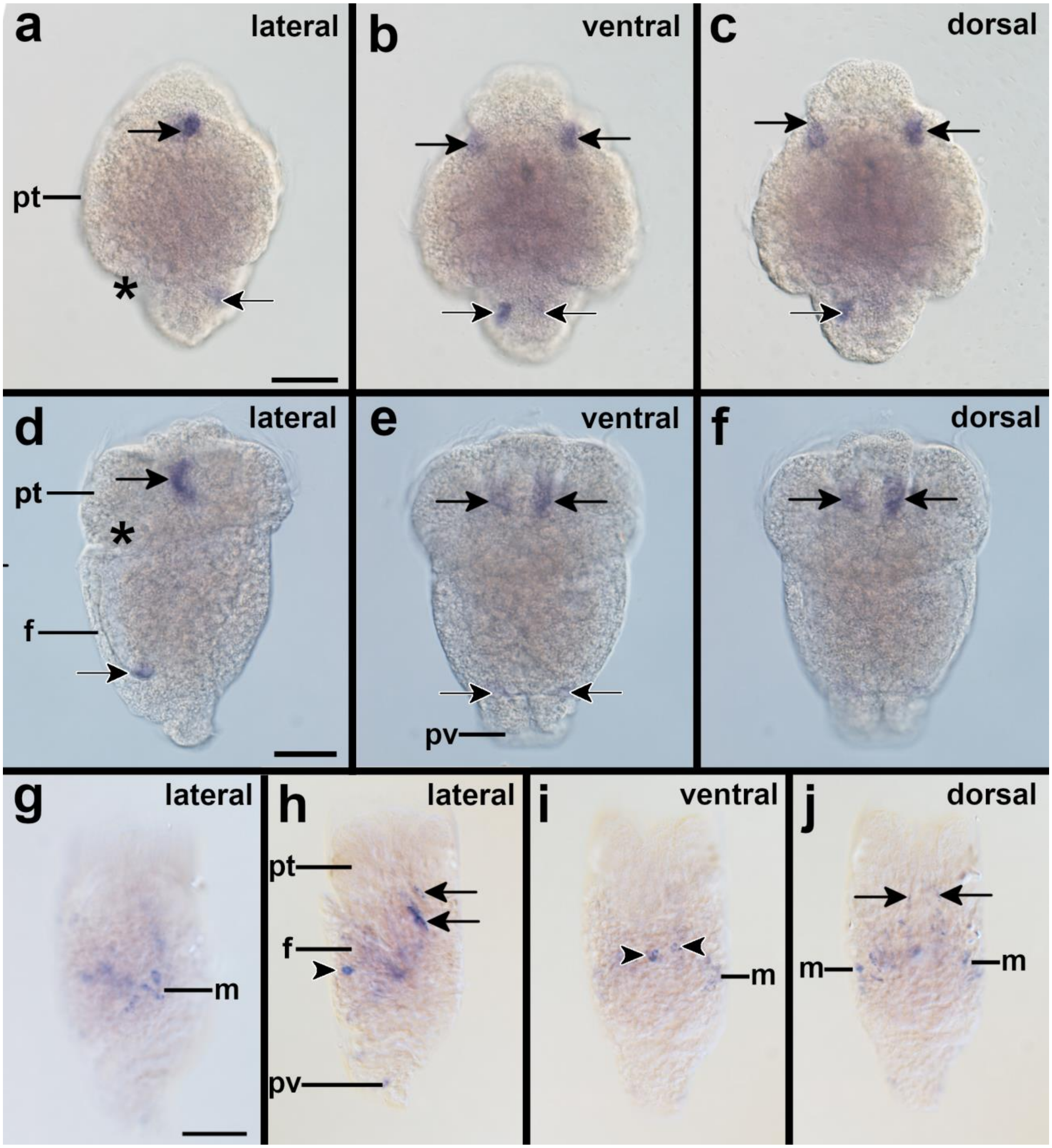

Figure S6. ***Pax6*** **expression during the development of the scaphopod** ***Antalis entalis*****.** Anterior faces up in all panels and dorsal faces to the right in lateral views. Asterisk labels the mouth. (**a-c)** Early trochophore larvae exhibit *pax6*+ cells in the region of the prospective foot (white-lined arrows). Another pair of *pax6*+ cells is situated in the cerebral pits, invaginations that have been documented to give rise to the cerebral ganglia precursors (black arrows). **(d-f)**

Early mid-stage trochophores exhibit two *pax6*+ cells in the posterior foot (white-lined arrows) and each two *pax6*+ flask-shaped cells in the lateral apical organ (arrows). **(g-j)** Latero-distal view **(g)**, latero-proximal view **(h)**, ventral view **(i)**, and dorsal view **(j)** of mid-stage trochophore larvae that expresses several *pax6*+ cells in the mantle, two *pax6*+ cells in the pavilion, two *pax6*+ cells in the anterior foot (white-lined arrowheads), and four *pax6*+ cells in the apical organ. The latter migrate in posterior direction into the trochophore larva (black arrows). Abbreviations: f foot, m mantle, pt prototroch, pv pavilion. Scale bars: 50 µm.

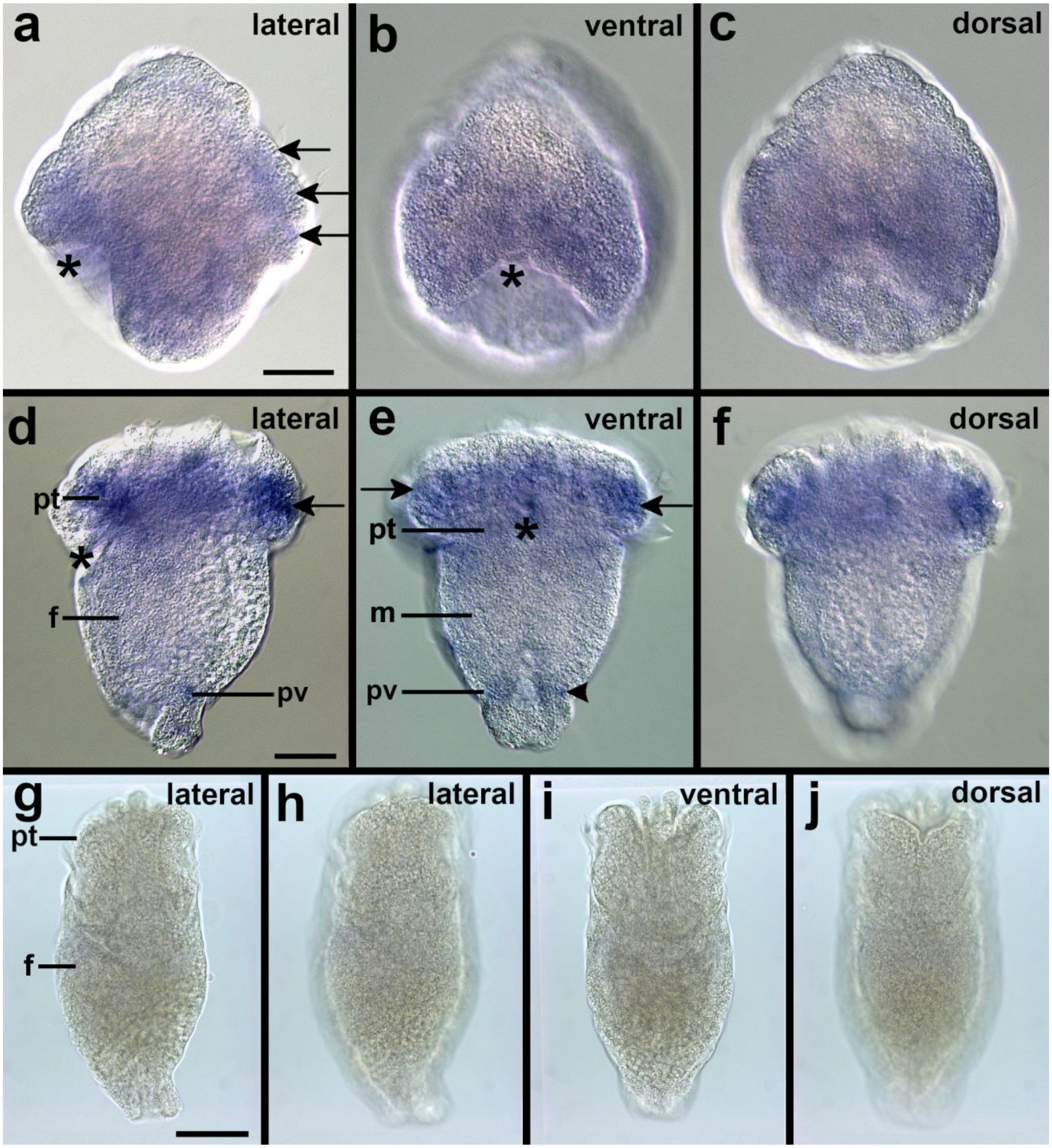

Figure S7. ***Rpgr*** **expression during the development of the scaphopod** ***Antalis entalis*****.**

Anterior faces up in all panels and dorsal faces to the right in lateral views. Asterisk labels the mouth. (**a-c)** Early trochophore larvae express *rpgr* in the trochoblasts (arrows). **(d-f)** Early mid-stage trochophores express rpgr in the trochoblasts (arrows) and in the pavilion (arrowhead). **(g-j)** Latero-distal view **(g)**, latero-proximal view **(h)**, ventral view **(i)**, and dorsal view **(j)** of mid-stage

trochophore larvae that possess no *rpgr*+ cells. Abbreviations: f foot, m mantle, pt prototroch, pv pavilion. Scale bars: 50 μm.

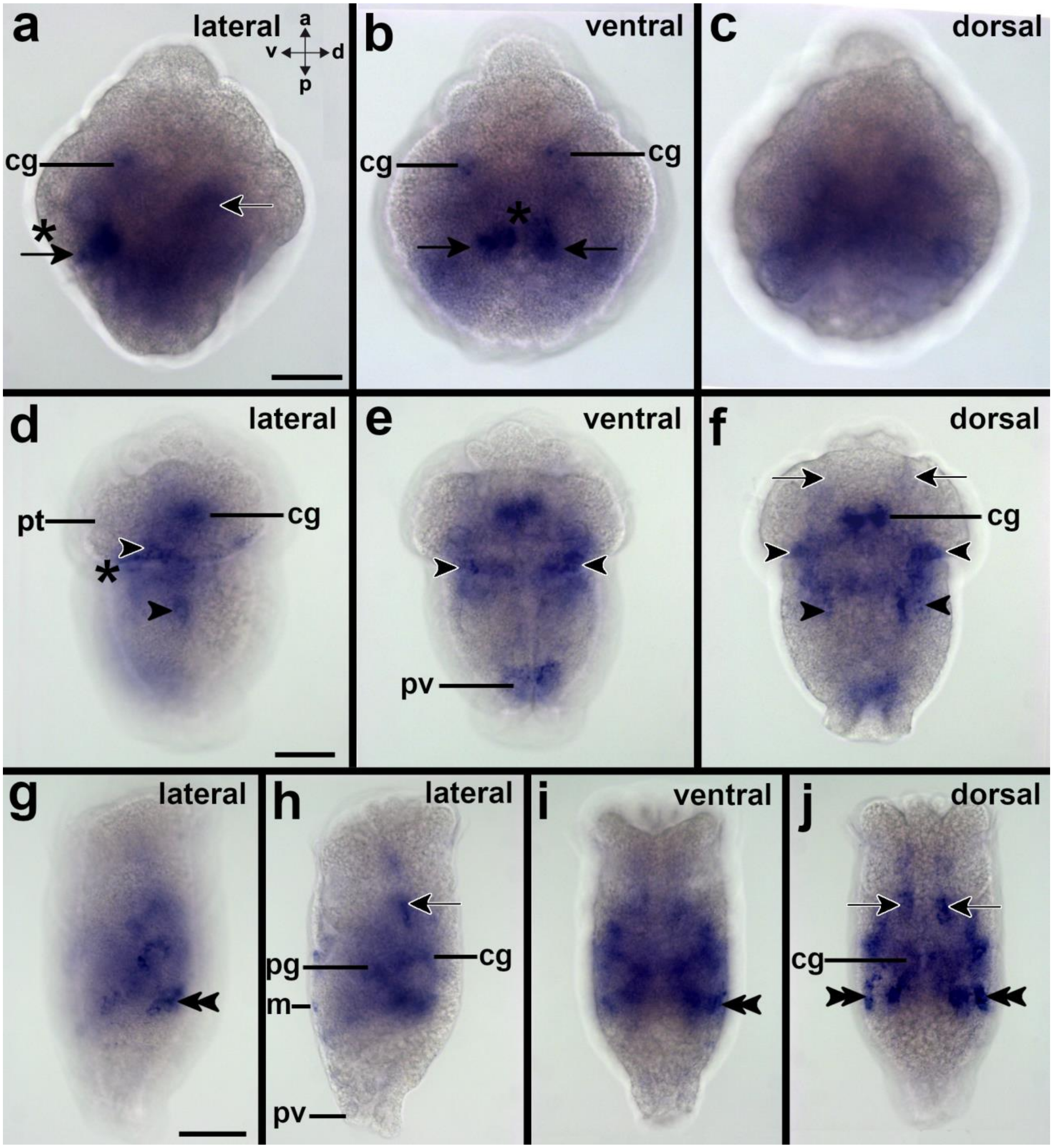

Figure S8. ***Six1/2*** **expression during the development of the scaphopod** ***Antalis entalis*****.**

Anterior faces up in all panels and dorsal faces to the right in lateral views. Asterisk labels the

mouth. (**a-c)** Early trochophore larvae express *six1/2* in two domains (black arrows) located in the region of the prospective foot. Additional expression is found in the tissue around the foregut in a region that could correspond to the anlagen of the cerebral ganglia and in two other cells that are located more posterior to the latter ones (white-lined arrow). **(d-f)** Early mid-stage trochophores express *six1/2* in the cerebral ganglia, on a low level in two cells in the apical organ (white-lined arrows), in cells in the mantle (black arrowheads), and in the region that connects the hyposphere with the episphere (white-lined arrowheads). **(g-j)** Latero-distal view **(g)**, latero-proximal view **(h)**, ventral view **(i)**, and dorsal view **(j)** of mid-stage trochophore larvae that expresses *six1/2* in two cells of the apical organ (white-lined arrows), in epidermal cells that are located dorso-laterally to the midgut gland (double arrowheads), in individual cells of the ventral mantle (m), and in cells of the prospective central nervous system, i.e. the cerebral ganglia (cg) and the pedal ganglia (pg). Abbreviations: cg cerebral ganglion, m mantle, pg pedal ganglion, pt prototroch, pv pavilion. Scale bars: 50 µm.

Table S1: Ancestral state reconstruction of larval apical and post-trochal photoreceptors suggesting that apical and post-trochal photoreceptors are molluscan synapomorphies.

	**Apical photore-ceptors**	***Go-opsin/ Xenopsin***	**Post-trochal photoreceptors**	***Go-opsin/ Xenopsin***	**Reference**
**Caudofoveata**	no evi-dence	Data missing	Data missing	Data miss-ing	Sigwart and Sumner-Rooney 2015
**Solenogastres**	no evi-dence	Data missing	No evidence	Data miss-ing	Todt and Wanninger 2010
**Poly-placophora**	Present	*Xenopsin*	present	*xenopsin*	Vöcking et al. 2015
**Cephalopoda**	no true larval stage (direct development)				
**Mono-placophora**	Data missing	Data missing	Data missing	Data miss-ing	
**Scaphopoda**	present	*go-opsin*	present	*go-opsin*	Present study
**Gastropoda**	present	Data missing	No evidence	Data miss-ing	Nielsen 2004
**Bivalvia**	present	Data missing	present	Data miss-ing	Nielsen 2004

No evidence: Taxon has been studied histologically and immunochemically but no photoreceptors were found.

References

Nielsen C. Trochophore larvae: cell-lineages, ciliary bands, and body regions. 1. Annelida and Mollusca.J Exp Zool.2004;302B:35-68.

Sigwart JD, Sumner-Rooney. Mollusca: Caudofoveata, Monoplacophora, Polyplacophora, Scaphopoda. In: Structure and evolution of invertebrate nervous systems. 2015. Eds. Schmidt-Rhaesa A, Harzsch S, Purschke G. Oxford University Press, Oxford.

Todt C, Wanninger A. Of test, trochs, shells, and spicules: Development of the basal mollusk *Wirenia argentea* (Solenogastres) and its bearing on the evolution of trochozoan larval key features.Front Zool.2010;7:6.

Vöcking O, Kourtesis I, Hausen H. Posterior eyespots in larval chitons have a molecular identity similar to anterior cerebral eyes in other bilaterians. EvoDevo.2015;6:40.

Sanger-sequenced nucleotide read of *go-opsin*:

TTCNTGTGTCCAATAGCATAGATAATTGGATTGTATGCACTTTGGGCCGTGGCAATTGC
TGTGGGTACTAGGGAGAGACCAAGCGGAATATATCCCCCAGCGGCAGATGCTAAAAAT
ATCAACCCGTATGGTATCCAAGCTATCACGTATACGACAATGGTAACCAGTATGATCTT
TGTAGCCCTTTTCCCAATATCTGGNATGTAGCTAACACCATGTCTTGAGTCGGTGATCT
GTGCCTTTGTTTGCGTGATACCATGGGACACTCTGACATGCNNNNTAACAAGGATCTT
CACGTACCAATATATCATAACGGTGAGTGGTAGGAACAATATGAAGATGACAACGCAT
ATTATGTAAGATACAGCTCCCTTGGNATGACCGTGCCAATCAATACTGCATGTCGTGCC
AAATGG

Predicted amino acid sequence (position 296 is in bold):

PFGTTCSIDWHGH?KGAVSYIICVVIFILFLPLTVMIYWYVKILV??HVRVSHGITQTKAQIT
DSRHGVSY?PDIGKRATKIILVTIVVYVIAWIPYGLIFLASAAGGYIPLGLSLVPTAIA**T**AQS
AYNPIIYAIGH

Methods

***Aen* Primer sequences used in the present study**

Primers used to amplify the region that translates into a T296 instead of a K296 residue.

Go-opsin forward primer: GAGGAATGGGGCTGCTCCAT

Go-opsin reverse primer: GACCTTGAACTTCATGTGTCCAATAGCATAG

Primers used to amplify template for riboprobe synthesis:

Go-opsin forward primer: CTATCACGTATACGACAATGGTAAC

Go-opsin reverse primer: CAATAAGTATGTACAGATACGCCGTG

Dach forward primer: ACAACTCATCCGTCTACAGAACTT

Dach reverse primer: CTGATCGTCATCTTCATCCTCTGT

Eya forward primer: ACAGAGGTTGGCATTCGTTGCTG

Eya reverse primer: CATATACATTAGATTTACAGCCCGCTGC

MyoV forward primer: CTTTCCCATTTGATAATCATATCCATGC

MyoV reverse primer: GATGCTCTTGATTTAATTGCTGTTAAAC

Pax6 forward primer: AAGGTCACAAAGATTTGCTACCGGAGGGC

Pax6 reverse primer: CGAAACAGATGAACAGATGCGCATGCGTC

RPGR forward primer: CTCAAATGATTTGATAACTTTTTATGATCTTCATC

RPGR reverse primer: GAGAGGGTAATAATGGTCAGCTTG

Six1/2 forward primer: GACGATGGCAGGGTACATG

Six1/2 reverse primer: GTCTGGTAACTACTAAGAGCTGAC

TrypC forward primer: CTGTACCAGACCAGGACCAATAC

TrypC reverse primer: CATGACAGTGTCTCTGTAAACATCTG

Xenopsin forward primer: GTTCCAGAGAGGGTACTGGAATG

Xenopsin reverse primer: CTTGGACGCCGTATGCAATAG

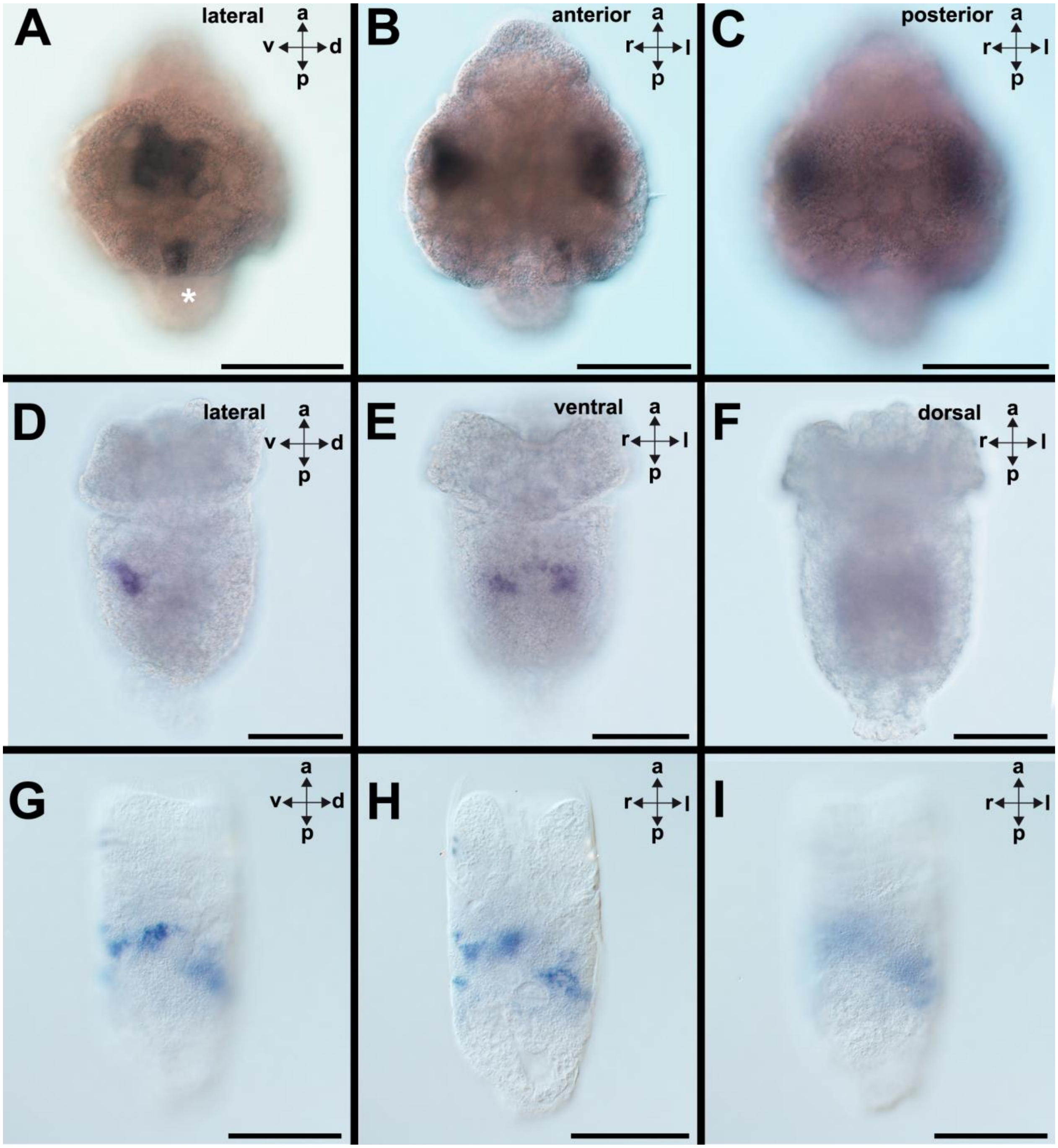

Figure S9. ***Hox4*** **expression during the development of the scaphopod** ***Antalis entalis*** **as off-target control for the** ***in situ*** **hybridization experiments.** Anterior faces up in all panels and dorsal faces to the right in lateral views. (**a-c)** Early trochophore larvae. **(d-f)** Early mid-stage trochophores **(g-j)** Mid-stage trochophores. Scale bars: 100 µm.
